# Liver-specific delivery of spherical DNA frameworks for alleviation of hepatic ischemic reperfusion injury

**DOI:** 10.1186/s12951-024-02661-8

**Published:** 2024-07-04

**Authors:** Hao Wang, Li Wen, Hao Wei, Yangmeihui Song, Wenyu Song, Mengting Li, Xiaoli Lan, Weibo Cai, Dawei Jiang

**Affiliations:** 1 Department of Nuclear Medicine, Tongji Medical College, Huazhong University of Science and Technology, 1277 Jiefang Ave, Wuhan, 430022 China; 2 Departments of Radiology and Medical Physics, Madison, WI 53705 USA; 3 Hubei Key Laboratory of Molecular Imaging, 1277 Jiefang Ave, Wuhan, 430022 China; 4 Key Laboratory of Biological Targeted Therapy, Ministry of Education, 1277 Jiefang Ave, Wuhan, 430022 China; 5 600 Highland Ave, K6/562, Madison, WI, 53792 USA

**Keywords:** Spherical DNA frameworks, Structure-activity relationship investigation, Hepatic ischemic reperfusion injury, Antioxidant

## Abstract

DNA nanostructures have long been developed for biomedical purposes, but their controlled delivery in vivo proposes a major challenge for disease theranostics. We previously reported that DNA nanostructures on the scales of tens and hundreds nanometers showed preferential renal excretion or kidney retention, allowing for sensitive evaluation and effective protection of kidney function, in response to events such as unilateral ureter obstruction or acute kidney injury. Encouraged by the positive results, we redirected our focus to the liver, specifically targeting organs noticeably lacking DNA materials, to explore the interaction between DNA nanostructures and the liver. Through PET imaging, we identified SDF and M13 as DNA nanostructures exhibiting significant accumulation in the liver among numerous candidates. Initially, we investigated and assessed their biodistribution, toxicity, and immunogenicity in healthy mice, establishing the structure-function relationship of DNA nanostructures in the normal murine. Subsequently, we employed a mouse model of liver ischemia-reperfusion injury (IRI) to validate the nano-bio interactions of SDF and M13 under more challenging pathological conditions. M13 not only exacerbated hepatic oxidative injury but also elevated local apoptosis levels. In contrast, SDF demonstrated remarkable ability to scavenge oxidative responses in the liver, thereby mitigating hepatocyte injury. These compelling results underscore the potential of SDF as a promising therapeutic agent for liver-related conditions. This aimed to elucidate their roles and mechanisms in liver injury, providing a new perspective for the biomedical applications of DNA nanostructures.

**Graphical Abstract:**

**Supplementary Information:**

The online version contains supplementary material available at 10.1186/s12951-024-02661-8.

**Figure:**
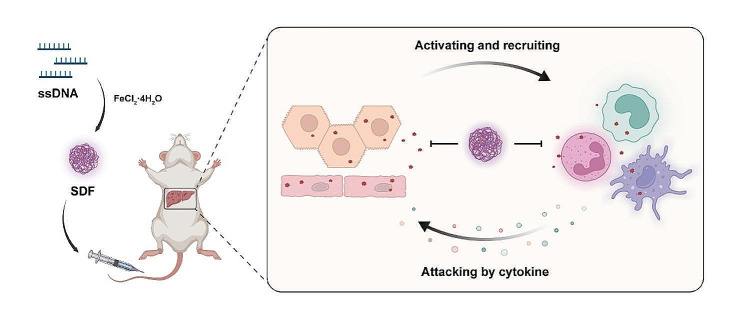
Spherical DNA framework showed predominant liver retention to rescue hepatic 870 ischemia reperfusion injury

## Introduction

Since the groundbreaking concept of utilizing DNA as a foundation for constructing intricate and programmable structures on the nano and micro scales [1–4], the realm of DNA nanotechnology has experienced remarkable growth, catalyzing advancements in chemistry, biology, photonics, and particularly, biomedicine [5–8]. DNA nanostructures, with their inherent biogenic nature and exceptional programmability, hold immense potential in biomedical research [9–13]. In 2012, Anderson and colleagues presented compelling evidence of DNA tetrahedrons modified with folic acids (FA) in detecting and imaging FA-receptor positive oral cancer [14]. Since then, DNA frameworks were used as versatile delivery platforms of fluorescent dyes, quantum dots, and chemotherapy drugs [15–18].

Recent studies have shown that DNA nanomaterials can be designed to target specific organs and tissues in the body, especially the kidney [19, 20]. In the 1990s, researchers reported major urinary excretion of radiolabeled single-stranded and double-strand DNA after systemic administration [21–23]. Through dynamic positron emission tomography (PET) imaging, we found that DNA tetrahedral frameworks at the sizes of 10 nm showed predominant renal excretions in mice, enabling accurate diagnosis of split kidney functions via renal PET [24, 25]. In addition, DNA origami nanostructures at 100 nm scales demonstrate concentrated accumulation in the kidneys, providing potent protection against acute kidney injury in mice [26]. 3D DNA origami frameworks performed differently in pharmacokinetics and bio-distributions, showing generally lower kidney accumulation but higher liver retention, compared with the reported sister structures (tFNA, 2D sheets and 1D rod). Even though, different frameworks presented distinct in vivo metabolism characteristics, including the duration time and accumulated concentrations in different organs. Outer surface modifications could further influence the structures’ behavior, such as exposed ssDNA would increase, while PEG moiety would decrease the liver accumulation of the 3D DNA, but the impact was also structure dependent [4].

Based on previous research on “nano-kidney”, we pose the critical question: What is the “structure-activity relationship” governing the in vivo biological distribution of various DNA nanostructures? Curiously intriguing, few studies have explored the targeted delivery of DNA nanostructures to the liver, a vital organ responsible for processing and metabolizing “foreign” substances [27, 28]. Studying how DNA nanostructures are processed and cleared by the liver is crucial for predicting their biodistribution, pharmacokinetics, and potential accumulation in hepatic tissues. This knowledge forms the foundation for achieving precise drug delivery and therapeutic strategies. Additionally, the liver, being a major immune organ, may influence the immune responses triggered by these nanostructures. Understanding these interactions is essential to minimize challenges related to immunity and ensure the development of biocompatible nanostructures. The notable absence of DNA nanomaterials within the liver, or the reticuloendothelial system in a broader sense, presents a challenge to our existing understanding of nano-bio interactions. This interesting observation also opens up new possibilities and prompts us to consider novel aspects of the “structure-activity relationship” unique to DNA-based nanocomplexes.

Bearing this notion in mind, we aim to test a number of DNA nanostructures and perform PET imaging to screen for liver-specific DNA frameworks. Among the nanostructures investigated, spherical DNA frameworks (SDF) and M13, a single-stranded circular virus genome spanning over 7000 nucleotides [26], emerged as two prominent candidates exhibiting significant accumulation within the liver following intravenous administration. To gain deeper insights into their structure-activity relationship in vivo, we explored their biodistribution profiles, assessed their toxicity and immunogenicity in healthy mice. Moreover, we sought to validate the nano-bio interactions of SDF and M13 in more challenging pathological conditions by utilizing murine models of liver ischemia-reperfusion injury (IRI), a commonly used animal model of acute liver injury and an often-seen clinical complication when performing liver surgeries. By subjecting the DNA nanostructures to this demanding pathological context, we aimed to gain a comprehensive understanding of their behavior and performance in the presence of liver damage, with the hope of shedding new light in biomedical applications of framework nucleic acids and liver disease theranostics.

## Results

### Preparation and radiolabeling of DNA frameworks

To identify nucleic acid frameworks that specifically target the liver, we prepared a series of DNA nanocomplexes and DNA strand controls, including single-stranded DNA (ssDNA, 20 nt), M13 circular DNA (M13, 7148 nt), tetrahedral DNA framework (TDF), bipyramidal DNA framework (BDF), and spherical DNA framework (SDF). TDF and BDF can be prepared by mixing and annealing a specific set of ssDNAs with designated sequences in the annealing buffer. The mixture was subjected to a temperature reduction from 95 ℃ to 4℃ over a period of 20 min, resulting in facile and stable formation of TDF and BDF, respectively [20, 24, 25, 29]. Gel electrophoresis analysis confirmed the successful preparation of these frameworks (Fig. S1A, Fig. S1B). Furthermore, dynamic light scattering measurements were performed to determine the size of TDF and BDF, which were found to be approximately 16.6 ± 2.6 and 26.9 ± 2.5 nm, respectively (Fig. S1C, Fig. S1D).

**Fig. 1:**
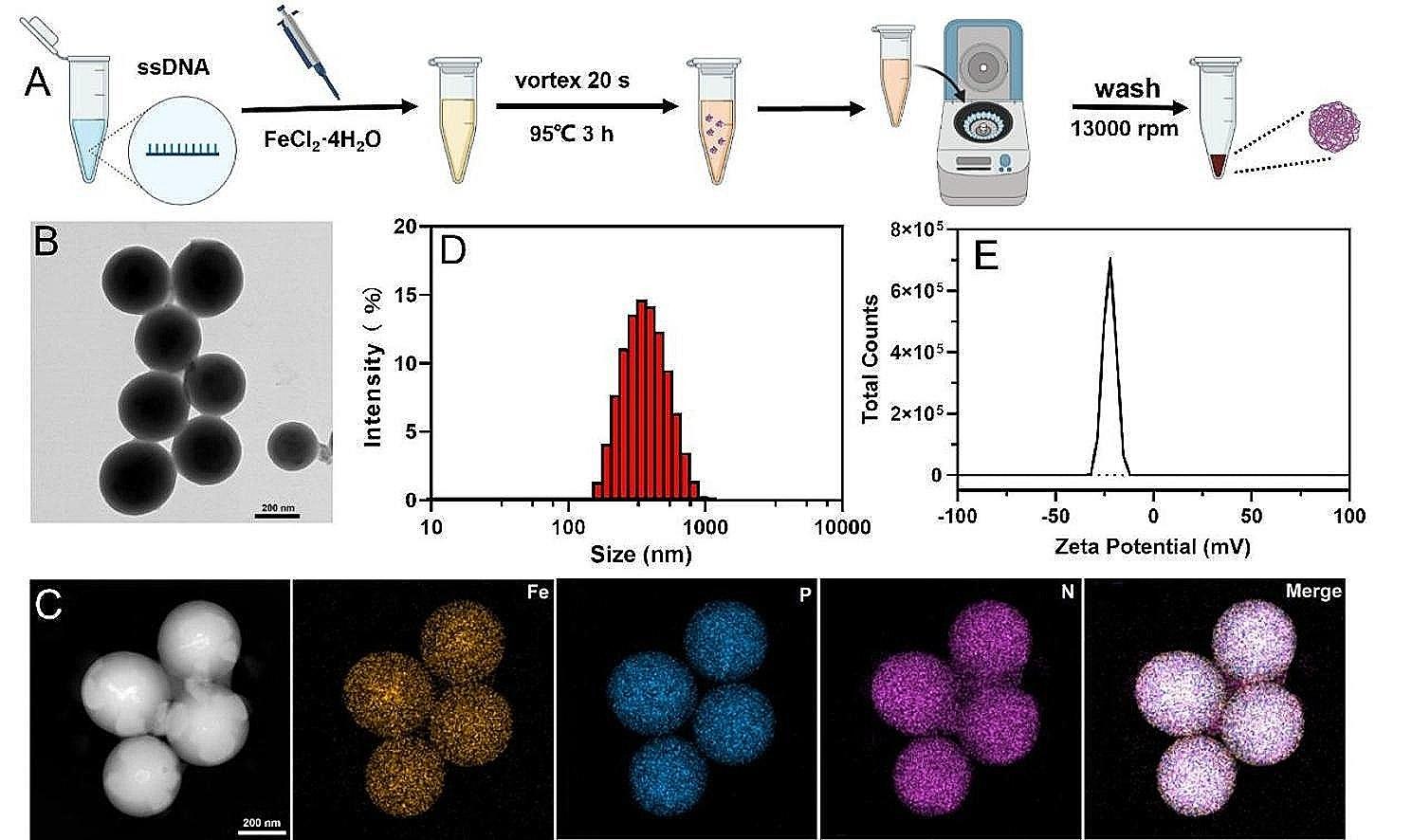
Preparation and characterizations of SDF. **A** Synthetic procedure of SDF. **B** TEM images of the synthesized SDF. **C** Elemental mapping of SDF, **D** Size distribution and Zeta potential (**E**) of SDF analyzed by dynamic laser scattering (DLS). scale bar: 200 nm

SDF was prepared by incubating a set of ssDNA with Fe^2+^ ions in an aqueous solution [30]. Following heating at 95 ℃ for 3 h and gradual cooling to room temperature, SDF can be obtained through centrifugation as white or light-yellow precipitates (Fig. 1A). The successful synthesis of SDF was confirmed by transmission electron microscopy (TEM) and elemental mapping (Fig. 1C). As can be seen from Fig. 1B, the obtained SDF showed spherical morphology with excellent dispersion in aqueous solutions. Dynamic light scattering measured its average hydrodynamic diameter to be around 309 ± 26.4 nm (Fig. 1D).

To label these DNA frameworks, Ga-68 (decay half-life 68 min) was used for in vivo PET imaging. An ssDNA complementary to DNA frameworks was conjugated with a NOTA chelator, followed by incubation with Ga-68 to obtain radiolabeled ssDNA, which was then hybridized with different DNA nanocomposites to produce radiolabeled DNA frameworks. The radiolabeling yield of ssDNA was measured to be over 70% for Ga-68, while the final DNA frameworks had specific activity in the range of 0.1-1 mCi/nmol after purification. SDF was labeled by incubating with free Ga-68 at 95℃ for 15 min. The spherical DNA aggregate will absorb Ga radiometals with a labeling yield of ~ 60%. In addition, in order to monitor the SDF in vivo for a longer time, we labeled the SDF with Cu-64 using the similar labeling method as Ga-68. All radiolabeled DNA frameworks were purified by PD-10 desalting columns and were used for subsequent PET imaging.

### SDF shows persistent liver accumulation via PET imaging

After radiolabeling of different DNA composites, we set forth to investigate their biological profiles in healthy mice via PET imaging, with the aim to screening for liver-specific structures. PET imaging of 20 nt ssDNA in healthy mice showed its quick excretion through the kidneys to the bladder. Blood circulation half-life of ssDNA was measured to be ~ 10.2 min. TDF and BDF underwent similar renal excretion with minimal retention in the liver or other major organs in vivo (Fig. S2). Bigger sizes of TDF and BDF lead to extended blood circulation of ~ 11.4 and 23.8 min, respectively. The half-life was obtained by fitting the blood pool time-activity curve with a biexponential model. A two-compartment fitting model was used to determine the half-life of ^68^Ga-TDF, ^68^Ga-BDF in blood.

Unlike ssDNA or DNA polyhedrons, M13 showed predominant liver uptake [26]. Similar to our previous work PET imaging of M13 showed rapid liver sequestration after intravenous injection (i.v.), with peak hepatic retention at ~ 20% injected dose per gram tissue (%ID/g). As time went by, M13 circular DNA strands were digested by the liver as evidenced by increased intestinal signal in PET images at later time points, where liver uptake dropped down from 20.3 ± 0.3%/ID/g at 3 h p.i. to 6.3 ± 0.3%/ID/g at 24 h p.i., at which most radioactivity was found in the gastrointestinal tract and feces.

**Fig. 2:**
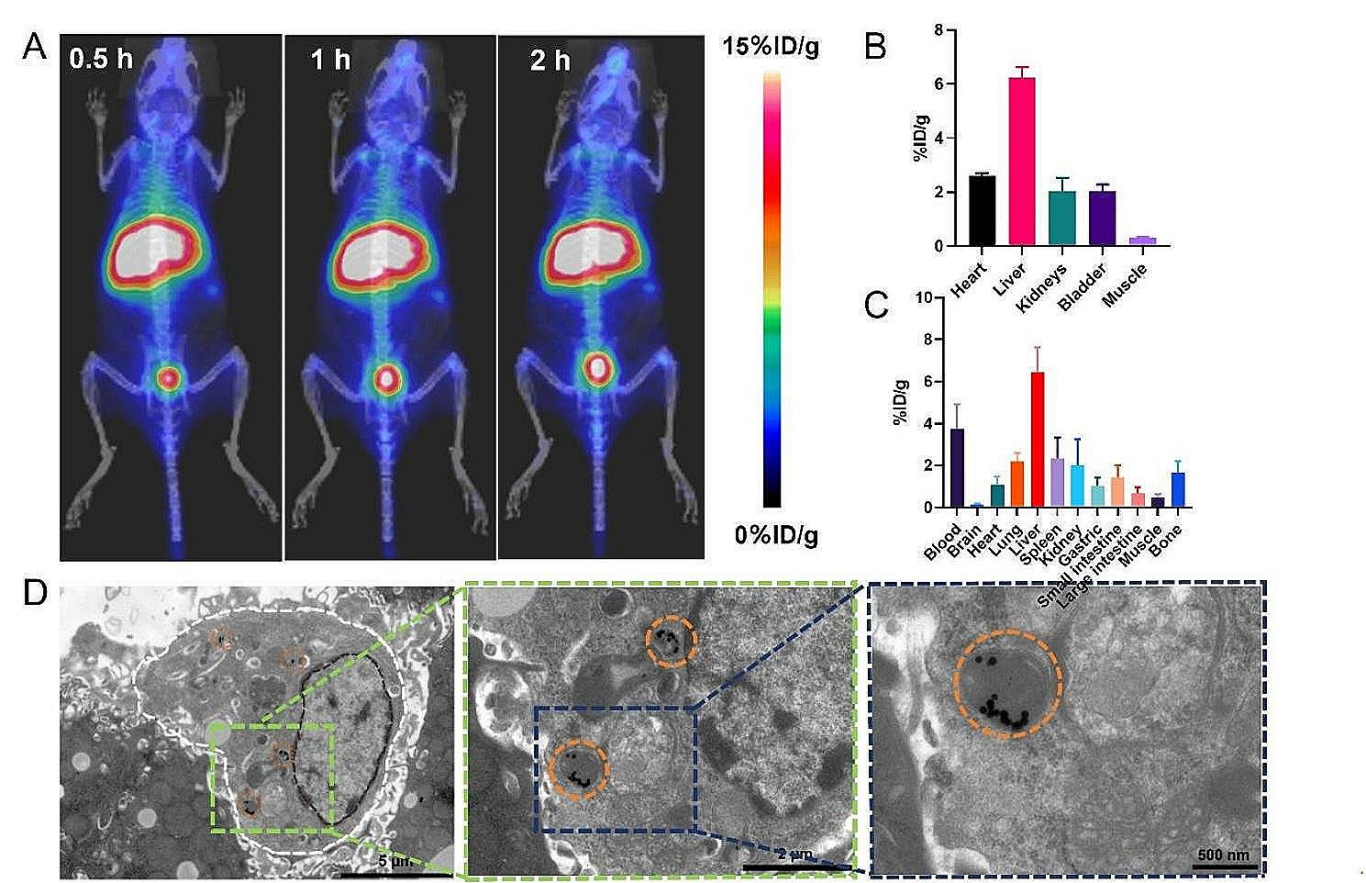
Biodistribution of SDF *in vivo.***A** Representative maximum intensity projection PET images of mice at various time points after i.v. injection of ^68^Ga-SDF. **B** ROI analysis of ^68^Ga-SDF PET images in healthy mice after i.v. injection 0.5 h. **C** Bio-distribution of ^68^Ga-SDF uptake in the blood, brain, heart, lung, liver, spleen, kidney, gastric, intestine, muscle, and bone at 2 h. **D** Bio-TEM images of mouse liver, the white line highlights the outline of Kupffer-like cells, the orange circle showed the endocytosis of SDF by lysosome. In (B, C) data represent means ± s.d. from three independent replicates

Similar to M13, PET imaging of SDF exhibited specific and persistent liver accumulation after administrating into healthy mice (Fig. 2A). Steady liver uptake was seen at 0.5, 1, and 2 h after ^68^Ga-SDF injection, with uptake values of 6.3 ± 0.3%/ID/g at 0.5 h and 6.4 ± 1.1%/ID/g at 2 h (Fig. 2B, Fig. S3). Biodistribution study confirmed the region-of interest (ROI) analysis of PET images (Fig. 2C). In addition, fluorescence imaging of SDF also showed specific and persistent hepatic accumulation (Fig. S4). To determine the metabolic fate of SDF in extended time, Cu-64 (decay half-life 12.7 h) labeled SDF was prepared and PET imaging demonstrated its consistent liver retention over a course of 24 h (Fig. S5).

### SDF has excellent biocompatibility

As a therapeutic agent for treating IRI, exploring the biocompatibility of SDF itself is imperative. Initially, we assessed the cytotoxicity of SDF in vitro, as depicted in **Fig S6**, where the cell survival rates consistently exceeded 85%. Subsequently, we administered SDF (1 mg/kg) to healthy mice and analyzed blood and organ samples to evaluate its biotoxicity. As illustrated in Fig. S7, H&E staining of organs in the SDF group revealed no significant damage compared to the PBS group. Furthermore, all hematological parameters remained within normal limits, there was no significant trend of weight loss and renal function indices in the SDF group closely resembled those in the PBS group. These findings collectively affirm the exceptional biocompatibility of SDF and its absence of biotoxicity.

### SDF targets kupffer cells with minimal liver toxicity

Having confirmed the preferential retention of SDF in the liver, biological TEM (bio-TEM) imaging was performed to identify detailed distribution of the structure on the cellular and sub-cellular level. A group of healthy mice was injected with SDF via the tail vein. At 1 h after injection, liver tissues were harvested, fixed, sectioned, and negatively stained for bio-TEM imaging. As shown in Fig. 2D, injected SDF was found phagocytosed in the lysosomes of Kupffer cells, in accordance with its sustained liver uptake found in PET images.

**Fig. 3:**
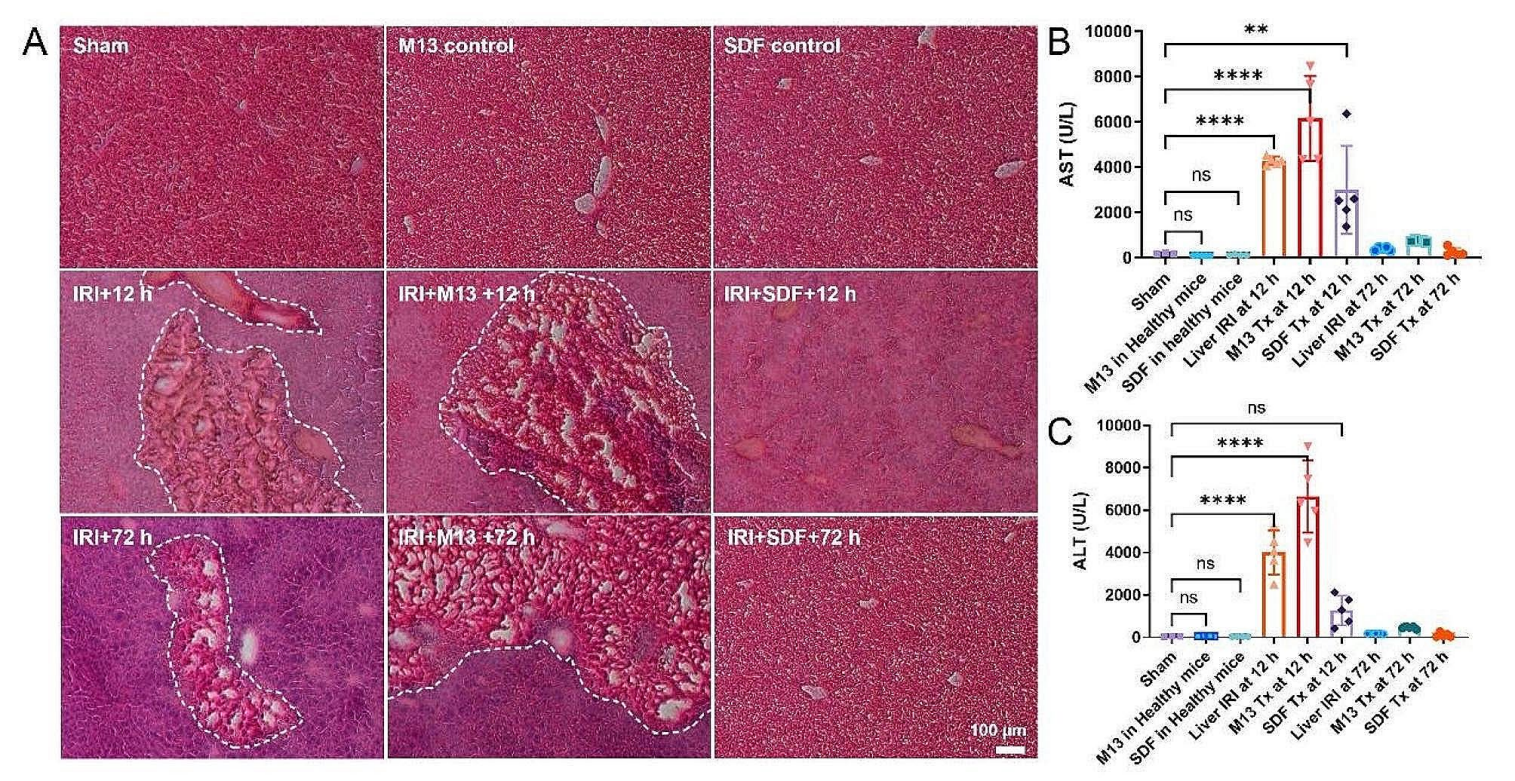
H&E staining and blood serum test after treatment of hepatic IRI. **A** H&E staining of liver tissues from each group. White dash line indicates the hemorrhage as well as cytolysis and necrosis of hepatic cells. Scale bar: 100 μm. **B, C** AST (**B**) and ALT (**C**) levels in blood serum from each group. Lower AST and ALT levels indicate better liver functions. In (**B, C**) data represent means ± s.d. from five independent replicates, and Pvalues were calculated by one-way ANOVA with Tukey’s honest significant difference post-hoc test (*P < 0.05; **P < 0.01; ***P < 0.001; ****P < 0.0001; ns, non-significant)

To examine potential toxicity of SDF on liver function, we performed comprehensive blood and immunogenicity tests in healthy mice. Blood tests on liver function, aspartate aminotransferase (AST) and alanine aminotransferase (ALT) revealed that no acute physiological changes were induced by SDF’s accumulation in the liver (Fig. 3B and C). Same blood test results were seen after injection of M13, suggesting overall biocompatibility of DNA frameworks after delivered to the liver (Fig. 3B and C). For immunogenicity concerns on systemic administration of DNA complexes, including interferon gamma (IFN-γ), tumor necrosis factor-α (TNF-α), interleukin-1 (IL-1), interleukin-6 (IL-6), and interleukin-12 (IL-12) were measured at 24 h after injection of either SDF or M13 (Fig. 5). M13 injection into healthy mice did exhibit mild immune stress as indicated by elevated levels of IFN-γ, TNF-α and down-regulation of IL-1, the downregulation of NOS2 levels and increase in MPO confirmed that M13 induced a mild oxidative imbalance in the liver. However, minimal toxicity was seen in healthy mice after i.v. injection of SDF.

GO analysis of potential target genes was performed using the DAVID database. Target genes were mostly enriched in complement activation, humoral immune response, and humoral immune response mediated by circulating immunoglobulin in BP enrichment analysis; immunoglobulin complex, circulating, immunoglobulin complex, and high-density lipoprotein particle in CC analysis; and immunoglobulin receptor binding, antigen binding, and endopeptidase inhibitor activity in MF analysis (Fig. S8A). The result of KEGG pathway enrichment analysis indicated that target genes were significantly enriched in complement and coagulation cascades, coronavirus disesse-COVID-19, and staphylococcus aureus infection (Fig. S8B). Both GO analysis and KEGG pathway enrichment analysis involved complement, the majority of which is synthesized in the liver. In addition, the most abundant albumin and apolipoproteins were also synthesized in the liver by SDF surface protein analysis (Fig. S8C). Both pathway analysis and surface protein analysis indicated the targeting of SDF to the liver. In addition, as shown in Fig.S9 we investigated the pharmacokinetics of SDF.

### SDF neutralizes ROS in *vitro*

The occurrence and progression of ischemia-reperfusion injury (IRI) are associated with local cellular damage caused by reactive oxygen species (ROS). Since DNA bases can be oxidized after their interaction with various types of ROS, DNA-based nanocomplexes can act as antioxidants as verified in our previous studies [13, 26]. We aimed to investigate whether the transport of M13 and SDF to the liver possesses antioxidative capabilities, eliminate reactive oxygen species, and ultimately alleviate the clinical symptoms of IRI. To explore this, we conducted in vitro antioxidation experiments with M13 and SDF to validate their capacity to eliminate ROS.

We performed assays with hydroxyl radicals (•OH) and superoxide radicals (•O_2_^−^), two typical types of ROS commonly seen in living organisms after inflammation or ischemic reperfusion injury. Results showed that both DNA materials (M13 and SDF) readily reacted with both types of ROS and lowered the level of oxidative stress in vitro (Fig. S10). Their capacity to scavenge reactive oxygen species was found in a concentration-dependent manner. Consequently, we confirm M13 and SDF as antioxidative DNA nanostructures and move forward for further in vivo studies.

### SDF alleviates liver ischemic reperfusion injury

Having confirmed the dominant liver accumulation of SDF and M13 in healthy mice and their potent ROS neutralizing effects in vitro, we further administrated them to animals with liver IRI, with the aim to test their potential therapeutic effects. Murine models of hepatic IRI were established according to previously reported methods. SDF was introduced intravenously at 2 h before IRI surgery for potential protection of hepatic injury. M13 was employed as another treatment since, similar to SDF, PET imaging saw its liver retention and in vitro tests confirmed its ROS scavenging capacity.

At 2 h after SDF injection, mice were subject to liver IRI surgery and the portal triad was clamped for 60 min, followed by removal of blood vessel clamps, reperfusion injury was thus induced and treatment effects were monitored by blood tests and liver tissue staining. As shown in Fig. 3B and C, mice with hepatic injury displayed elevated blood levels of AST and ALT (> 4000 U/L), and H&E staining revealed obvious area of hepatocyte damage at 12 h after surgery, suggesting successful establishment of the animal model. Mice in the sham group, and healthy mice receiving SDF or M13 showed no sign of toxicity or abnormalities with regard to their liver function and microstructural morphology. In IRI mice receiving SDF treatment, AST and ALT levels dropped down to healthy ranges and liver H&E staining showed obviously less damage comparing to the PBS treatment group. Interestingly, M13 treatment induced increased liver damage in mice with hepatic IRI, as evidenced by elevated AST and ALT concentrations in the blood (even more than 6000 U/L), we could see from the white dashed area in Fig. 3 that there were large areas of severe damage accompanied by significant cytolysis, hepatocyte necrosis and hemorrhage in the liver sections of IRI mice as well as in the M13 treated IRI mice. Liver sections from M13 treated IRI mice, with multiple large cavities visible, more severe damage than the PBS treated IRI mice, indicated that the injection of M13 aggravated the degree of IRI, and significantly larger areas of hepatocyte injury all over the liver tissue slices.

We further analyzed treatment effects at 72 h after injection. Massive areas of necrosis were found in liver tissue sections from the M13 treated IRI group and PBS treated IRI group at 72 h, suggesting that the damage had exceeded the liver’s ability to repair itself. No significant damage was seen in the SDF treated IRI group after 72 h and H&E staining results were similar to those of the sham group and SDF injected groups in mice. Similar to results found at 12 h after inducing IRI injury, we saw that M13 aggravated hepatic IRI injury, and SDF could well prevent hepatocyte damage. Unexpectedly, at 72 h after the treatment, AST and ALT levels in each group dropped down, getting closer to healthy ranges, suggesting that the self-healing ability of mouse liver is stronger to our expectation and different from clinical observations of liver IRI injury in humans. Taken together, even through murine models of liver IRI showed signs of self-recovery, SDF still presented its role in in providing sustained protection to the liver.

### SDF inhibits oxidative stress to prevent liver IRI

**Fig. 4:**
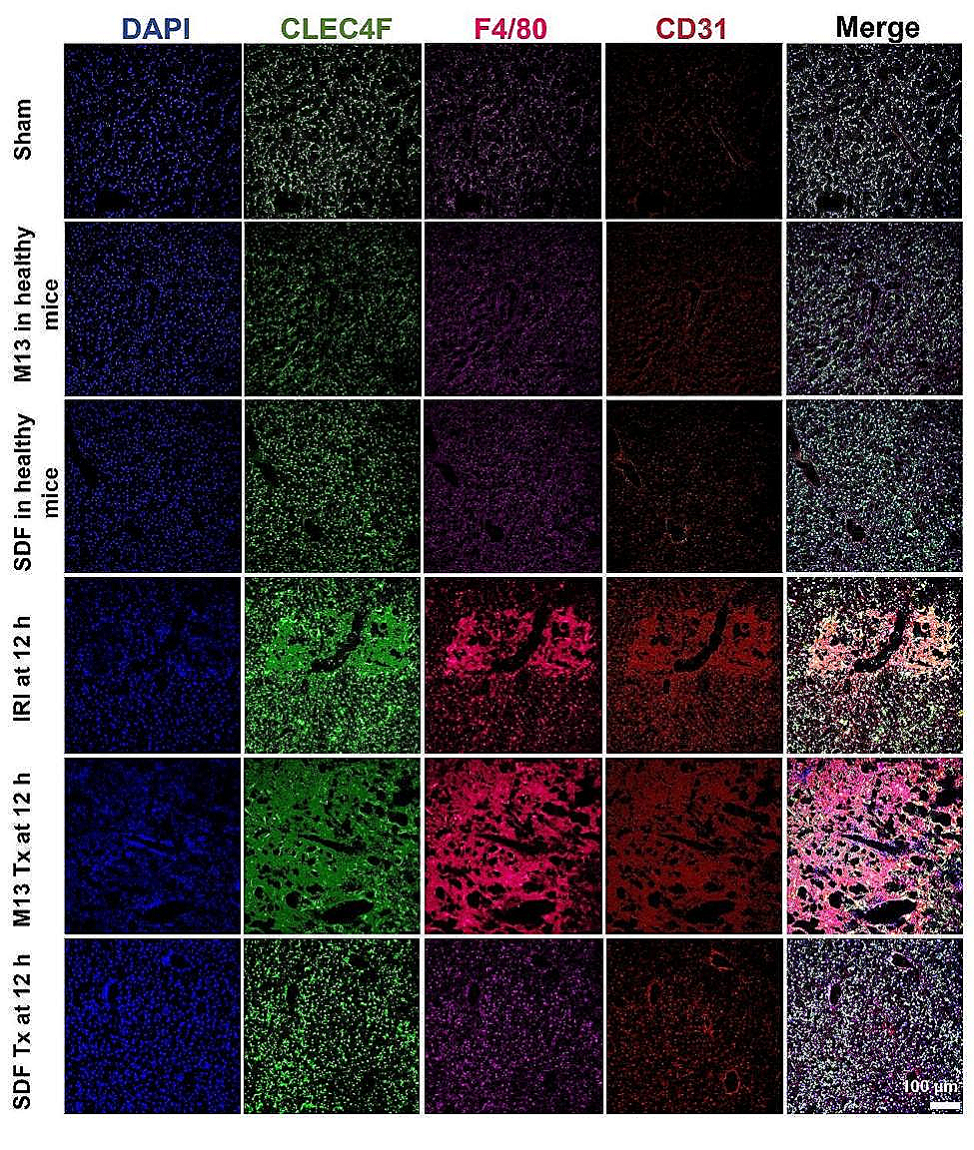
Immunofluorescence staining on liver samples. Immunofluorescence staining was performed using DAPI (blue) for nuclear staining, anti-CLE4F antibody (green) as Kupffer cell marker, anti-F4/80 antibody (pink) as monocyte/macrophage marker, anti-CD31 antibody (red) as an endothelial marker of liver tissues from each group. Scale bar: 100 μm

Oxidative stress is considered one of the major culprits of hepatic IRI. To demonstrate the role of cellular phenotype in the mechanism by which SDF prevents IRI, we performed immunofluorescence imaging of liver samples. DAPI, anti-C-Type Lectin Domain Family 4 Member F (anti-CLEC4F), anti-F4/80, and anti-CD31 antibodies were used for staining of the nuclei (blue), Kupffer cells (green), monocyte/macrophage cells (pink), and vascular endothelial cells (red), respectively. As shown in Fig. 4, after injection of M13 or SDF into healthy mice, there is no sign of Kupffer cell and monocyte activation as can be seen in the Sham group, indicating excellent biocompatibility of both DNA materials. In addition, CD31 staining in these groups confirmed intact endothelium integrity and minimal liver damage. In contrast, at 12 h after induction of liver injury, up-regulated and disrupted CLEC4F and F4/80 staining patterns (Fig. 4) demonstrated activation of Kupffer cells and monocytes, which accumulated around the injured sites in the liver though endothelium. Activated Kupffer cells would then release various cytokines, and produce ROS to further recruit monocytes/macrophages in a positive feedback loop that ultimately leads to hepatic ischemic reperfusion injury. When administrating liver-targeting DNA materials, M13 or SDF, bifurcated findings were seen in excellent accordance with blood test and H&E staining results. In M13 treated IRI group, Kupffer cells and monocytes are massively activated and integrity of endothelium is no longer intact, suggesting aggregated damage after injection of M13. In contrary, SDF treatment significantly suppressed Kupffer activation cascade as evidenced by regulated and in-diffusive staining of the endothelium, indicative of its treatment effect. Since SDF may scavenge excess ROS, minimal Kupffer cells, and macrophages in general, are recruited to preserve the endothelium in liver tissues after administration. Semi-quantification analysis of our confocal images further supports these findings (Fig. S11).

Neutrophils have been well recognized as one of the major effectors during acute injury, which can be recruited to the inflammatory site within minutes. We then evaluated the recruitment of neutrophils and hepatocyte apoptosis by immunofluorescence staining on liver samples. Intracellular adhesion molecule-1 (ICAM-1), an adhesion biomarker of neutrophils, was stained together with Caspase-3 to inform immune recruitment and hepatocyte apoptosis, respectively. As shown in Fig. S12, healthy mice treated with either DNA materials and the sham group exhibited low ICAM-1 expression, intact endothelial integrity, and negligible hepatocyte apoptosis. However, hepatic IRI and M13 treatment showed elevated ICAM-1 expression, apoptosis, and compromised endothelial integrity. Notably, the SDF-treated group demonstrated a reversal of all these symptoms at 12 h after surgery. High expression of the ICAM-1 on the intraluminal side of liver sinusoidal endothelial cells is believed to contribute to the rolling, binding, and parenchymal extravasation of neutrophils. We further evaluated the infiltration of neutrophils using an anti-Ly6G antibody as the neutrophil marker. Fig. S13 showed significant neutrophils infiltration in IRI mice receiving M13 treatment. In comparison, SDF treated IRI animals exhibited minimal neutrophils infiltration at 12 h after surgery. Semi-quantification analysis of our confocal images further supports these findings (Fig. S11).

Immunofluorescence staining of liver tissues at 72 h after IRI induction were found similar to those at 12 h. In the M13 treated IRI group, a large number of Kupffer cells and monocytes were activated, endothelial integrity was impaired, neutrophils were recruited and infiltrated, and hepatocytes were apoptotic. On the contrary, these conditions were reversed after SDF treatment, validating that SDF could continuously and effectively eliminate reactive oxygen species and alleviate liver injury (Fig. S14, Fig. S15, Fig. S16). In addition, immunofluorescence staining results indicated that both M13 and SDF did not adversely affect the liver under physiological conditions. While the single-stranded circular DNA M13 could aggravate the degree of IRI under pathological conditions of hepatic ischemia-reperfusion injury, SDF could effectively navigate to the liver and alleviate the damage by scavanging ROS.

### Effect of inflammatory cytokines and peroxidase in hepatic IRI

**Fig. 5:**
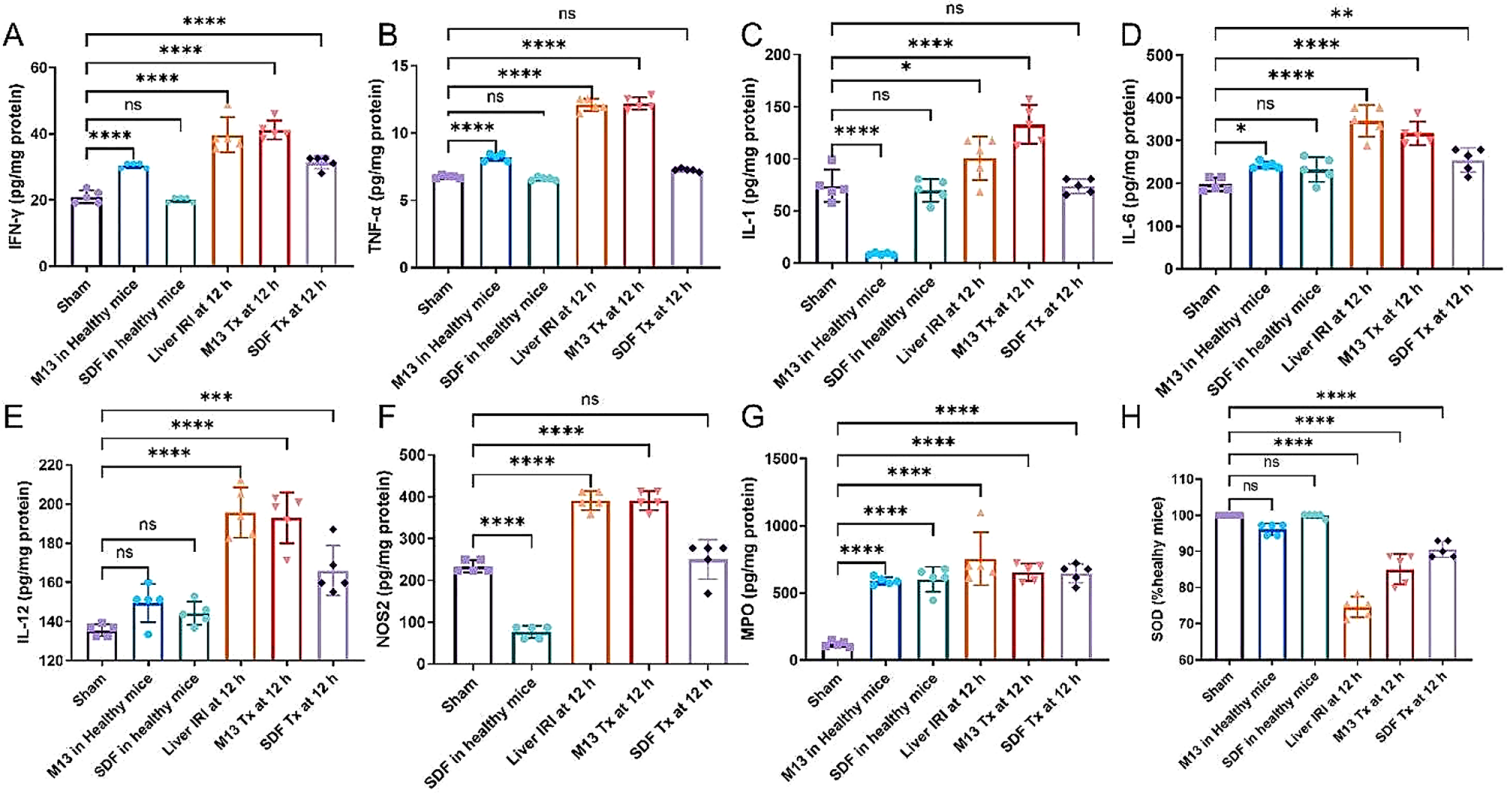
Detection of cytokines in liver tissues. **A-H**) Cytokines of IFN-γ (**A**), TNF-α (**B**), IL-1 (**C**), IL-6 (**D**), IL-12 (**E**), and NOS2 (**F**) from activated monocyte/macrophages and Kupffer cells and MPO (**G**) from activated neutrophil were measured in liver homogenates and SOD(**H**) form each group. Data represent means ± s.d. from five independent replicates, and P values were calculated by one-way ANOVA with Tukey’s honest significant difference post-hoc test (*P < 0.05; **P < 0.01; *** *P* < 0.001; **** *P* < 0.0001; ns, non-significant)

In the above study, we found significant activation of monocytes/macrophages and Kupffer cells. Previous reports have suggested that these activated immune cells can increase the release of ROS and pro-inflammatory cytokines, which act as cytotoxins on endothelial cells and hepatocytes, thereby inducing liver injury. To investigate the anti-inflammatory effects during hepatic IRI, we assessed various cytokines in liver homogenates, including IFN-γ, TNF-α, IL-1, IL-6, and IL-12. Our data clearly showed a significant increase in the levels of these pro-inflammatory cytokines in the IRI group, with a further elevation observed in the M13-treated group compared to the IRI group. In contrast, the SDF-treated IRI group exhibited reductions in these cytokine levels, approaching a relatively normal range. Of note, IL-1, known for its pro-inflammatory properties, not only enhances TNF-α production by Kupffer cells but also stimulates ROS release by neutrophils, the levels of IL-1 in the IRI group and the M13-treated group were significantly higher compared to the SDF-treated group, which approached normal levels. Similarly, TNF-α, as a pro-inflammatory cytokine, contributes to immune-inflammatory responses and promotes the activation of Kupffer cells and macrophages, the levels of TNF-α in both the IRI group and the M13-treated group showed a significant increase. Moreover, our results demonstrated that TNF-α upregulates IL-6, which serves as a stimulus for the innate immune response, as further supported by Fig. 5, in contrast to the heightened IL-6 expression observed in the IRI and M13-treated group, the SDF-treated group exhibited a downregulation of IL-6 levels, attributable to the antioxidative properties inherent in SDF. The upregulation of various pro-inflammatory cytokines also activates T helper cells and NK cells. IFN-γ, predominantly produced by activated Th cells and NK cells, rapidly recruits neutrophils to the site of inflammation, exacerbating the inflammatory response. IL-12, a cytokine produced by activated antigen-presenting cells, is vital for immune function, especially in Th1 cell differentiation and NK cell activation. Like IFN-γ, IL-12 levels were lower in sham surgery and mice injected with M13 and SDF. However, the IRI and M13-treated groups showed a noteworthy increase in IFN-γ and IL-12 compared to the SDF-treated group. In our study, liver homogenates from IRI mice and M13-treated IRI mice exhibited significant upregulation of nitric oxide synthase 2 (NOS2) expression due to inflammation, leading to excessive nitric oxide production and subsequent aggravation of liver injury. However, in mice treated with SDF, the expression of NOS2 was not evident, suggesting a potent protective effect against liver injury. Myeloperoxidase (MPO) is a peroxidase mainly expressed in neutrophils. As shown in Fig. 5, the level of MPO in IRI group was significantly increased, indicative of strong neutrophil recruitment. On the contrary, the levels of MPO in the livers of IRI mice treated with SDF, both at 12 and 72 h (Fig. S18) after surgery, retreated to healthy ranges, confirming that SDF can attenuate neutrophil recruitment to the liver, mitigate the hepatic immune response, and prevent liver injury. In addition, we measured superoxide dismutase (SOD) levels in liver homogenates of each group. SOD serves as an indicator of oxidative stress and plays a crucial role in scavenging oxygen radicals. The data revealed a significant reduction in SOD levels in the IRI group compared to the sham group and the groups receiving M13 or SDF injections in normal mice. In contrast, the SDF-treated group exhibited weaker oxidative stress, suggesting that SDF served as a reducing agent to neutralize ROS, restore SOD levels, and provide further protection to hepatocytes in IRI mice.

### SDF inhibits M1 and promotes M2 activation

**Fig. 6:**
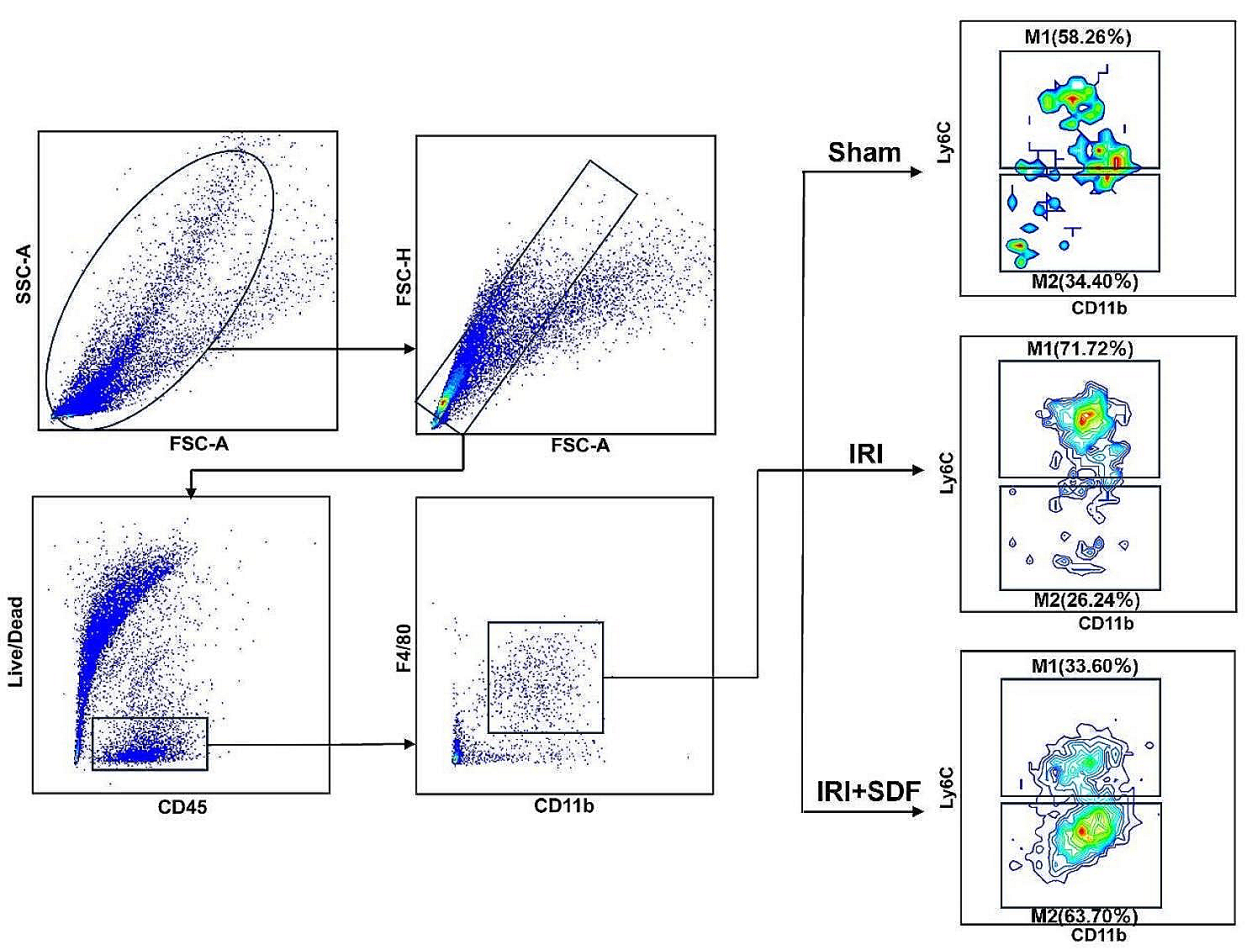
Flow cytometry was used to analyze the percentage of M1 and M2 in liver tissues in the three groups of Sham, IRI, and IRI + SDF

The successful inhibition of inflammatory cytokines by SDF has given us new mind for thought. Inflammatory cytokines are secreted by M1 macrophage [31], and whether SDF plays a role in the inhibition and promotion of macrophage differentiation. The role of the M1 macrophage is to secrete pro-inflammatory cytokines and chemokines, to present antigens, and thus to participate in an active immune response, functioning as an immune surveillance [31, 32]. The main pro-inflammatory cytokines it produces are IL-6, IL-12 and TNF-alpha [32]. M2 macrophage mainly secrete anti-inflammatory cytokines such as Arginase-I, IL-10 and TGF-beta, which have the function of reducing inflammation and promoting tumor growth and immunosuppression [33, 34]. It plays an important role in wound healing and tissue repair. We analyzed the effect of SDF on macrophages by flow cytometry. Firstly, the cell experiment, we divided the macrophage RAW264.7 into three groups, one group was normal without intervention group, the second group was the group adding Lipopolysaccharide (LPS, concentration of 100 ng/mL), which promoted the activation of M1, and the third group was the group adding LPS (100 ng/mL) and SDF (concentration of 20 ug/mL) together. As can be seen from Fig.S19, Fig.S20A, LPS successfully activated M1, and the proportion of M1 increased from about 15% to about 40% in the normal group, while the proportion of M1 was about 30% in the group with the presence of SDF, indicating that SDF could effectively inhibit the activation of M1. Secondly, the effect of SDF on macrophages was further determined by an in vitro HIRI model. The macrophage RAW264.7 was subjected to hypoxia for 12 h and reoxygenation for 6 h to create an in vitro HIRI model. As can be seen from Fig.S21, in the in vitro HIRI model, M1 was polarized and the percentage was elevated. the percentage of M1 increased from about 9.3% in the normal group to 40.2% in the IRI group, while the percentage of M1 in the experimental group with the addition of SDF was about 34.1%, which indicated that SDF could effectively inhibit the activation of M1. In the flow cytometry analysis for liver tissues (Fig. 6), the proportion of M1 in the IRI group was significantly higher than that in the Sham group, suggesting that MI was accelerated to activate after ischemia-reperfusion had occurred, resulting in the production of more pro-inflammatory cytokines, whereas in the SDF group, the proportion of M1 was significantly reduced from 71.7 to 33.6%, whereas the proportion of M2, which has anti-inflammatory effects, was significantly increased from 26.2 to 63.7%. In conclusion, SDF can effectively inhibit M1 and promote the activation of M2.

## Discussion

In this study, we successfully prepared, radiolabeled, and screened a set of DNA frameworks using PET imaging to identify liver-targeting nucleic acid nanostructures in healthy mice. Among tested DNA complexes, single-stranded DNA, tetrahedral and bipyramidal DNA frameworks showed preferential renal clearance, in excellent accordance with previous reports and validating kidney excretion threshold. On the other hand, spherical DNA framework (SDF) and the single-stranded circular DNA (M13) were found to undergo hepatic sequestration predominantly in vivo. While these two sets of DNA frameworks demonstrated opposite metabolic pathways and biological fates in vivo; toxicity examination, including blood tests, tissue staining, and liver/kidney function measurement, revealed no adverse effects after administration. Further validations are needed to systematically examine DNA structures’ acute and chronic toxicity to determine their median lethal dose (LD50), potential immunogenicity, and risk of genomic toxicity.

While it is excellent to identify kidney or liver targeting nanostructures for pertinent biomedical application development, it would be more intriguing to ponder the underlying mode of actions that guide these structures into specific metabolic pathways. Previously, we summarized how nanocomplexes may interact with the kidney for renal disease imaging and treatment [13, 19]. We categorized these nano-kidney interactions into two major types: renal excretion and renal accumulation [9, 20, 25]. However, in this study, our results suggests that hepatic interaction comes first and, to some extent, decides whether subsequent renal interactions may occur. This intuitive speculations from PET imaging observations highlight the need to investigate in details the potential theory on relationships between DNA nanostructure and liver activity.

**Scheme 1:**
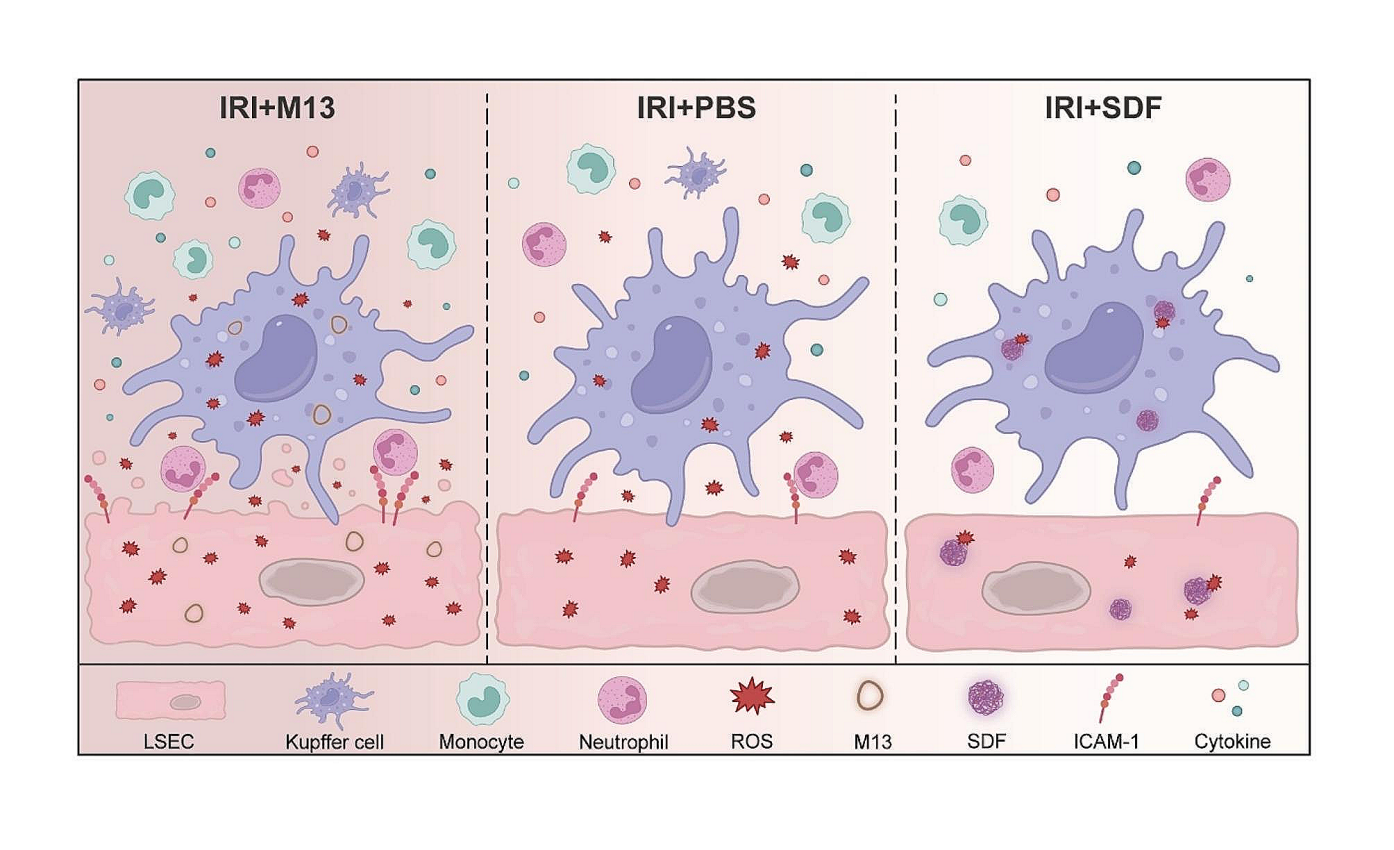
Mechanism of hepatic IRI prevention by nano-antioxidants. Compared with unintervened hepatic IRI, SDF effectively scavenged intracellular (e.g., hepatic sinusoidal endothelial cells and Kupffer cells) and extracellular ROS in the liver. With the neutralization of ROS, the activation of Kupffer and monocyte cells was reduced, and the release of pro-inflammatory cytokines was limited, which resulted in the inhibition of neutrophil recruitment and infiltration. Conversely, under hepatic IRI pathological conditions, M13 stimulates more ROS production, further enhancing the immune cascade response and exacerbating liver injury

Upon identifying liver-avid DNA frameworks in physiological conditions, we further challenged them with a more complex condition, namely murine models of liver ischemic reperfusion injury (Scheme 1**)**. Interestingly, our findings revealed significant therapeutic effects of SDF compared to M13. M13 not only exacerbated hepatic oxidative injury but also elevated local apoptosis levels, as evidenced by endothelial damage observed in liver tissue staining and increased interleukin secretion. Contrarily, SDF exhibited notable therapeutic efficacy. Histological analysis of H&E staining sections revealed absence of significant damage, alongside normalization of AST and ALT indices, and noteworthy reduction in inflammatory cytokine levels compared to the M13 group. SDF demonstrated capacity to neutralize ROS, suppress Kupffer and M1 macrophage activation, promotes activation of M2 macrophage, attenuate the release of inflammatory cytokines, and mitigate the adherence and infiltration of centriole granulocytes, thereby ameliorating hepatic inflammation and mitigating liver injury. These compelling results underscore the potential of SDF as a promising therapeutic agent for liver-related conditions.

## Conclusion

Through nuclear medicine imaging, we identified SDF from our prepared DNA framework capable of liver retention. In the pathological condition of IRI model, SDF effectively scavenges ROS. As ROS are cleared, the activation of Kupffer cells and monocytes decreases, limiting the release of pro-inflammatory cytokines, thereby inhibiting recruitment and infiltration of neutrophils, attenuating immune cascade reactions, and highlighting the promising potential of SDF as a therapeutic agent for liver-related diseases. Previously, the metabolism and fate of nucleic acid materials in the form of three-dimensional frameworks in vivo have not been systematically investigated, which has also limited the development of their pharmacologization and in vivo delivery. The results of this study provide new insights into the interactions between DNA frameworks and living organisms, and add to previous studies on the interactions between DNA frameworks and the liver, as well as a few thoughts on the potential theory regarding “structure-activity relationship” of DNA nanocomplexes. Different forms of DNA framework structures with different metabolic pathways with the potential to be enriched in different tissues are expected to serve as organ-targeting tools and provide new vectors for relevant disease medication. Exploring the detailed mechanism of action may promote the development of DNA nanostructure-based nanomedicine for biomedical applications.

## Materials and methods

### Materials

Iron chloride tetrahydrate (FeCl_2_·4H_2_O, 99%), Magnesium chloride (MgCl_2_), Sodium acetate (CH_3_COONa), Sodium bicarbonate (Na_2_CO_3_), and Sodium carbonate (NaHCO_3_) were purchased from Sigma-Aldrich. All chemicals were used as received without further purification. The following DNA strands were synthesized and purified by Sangon Biotech Co., Ltd. (Shanghai, China).

### Self-assembly of DNA frameworks (TDF, BDF, SDF)

The preparation method of TDF has been reported in the previous literature published [24, 25]. In brief, TDF was prepared by annealing the four DNA oligonucleotide strands (oligonucleotide sequences were shown in the Electronic Supplementary Material) in TM buffer (10 mM Tris-base, 50 mM MgCl_2_, pH 8.0) and rapidly cooling from 95 °C to 4 °C within 30 min to obtain the TDF.

The preparation method for BDF has been documented in our prior publication [20]. In summary, BDF was synthesized by annealing six DNA oligonucleotide strands (sequences provided in the Electronic Supplementary Material) in TM buffer (10 mM Tris-base, 50 mM MgCl_2_, pH 8.0). The annealing process involved rapid cooling from 95 °C to 4 °C within 30 min, resulting in the formation of BDF.

A fast one-step self-assembly method was used to prepare the SDF [30]. In brief, an aqueous solution of FeCl_2_·4H_2_O (20 mM, 30 µL) was added to an aqueous solution of DNA (25 µM, 570 µL) in a 2 mL PCR tube. After blending for 20 s, the mixture was placed on a metal bath and left to react for 3 h at 95℃. After the reaction was completed, remove the tube and allow it to cool down to room temperature, then wash three times by centrifugation with deionized water at 13,000 rpm. Finally, the synthesized SDFs were treated with a 0.45 μm filter membrane prior to the experiments to ensure that the SDFs used in the experiments were uniformly sized, the obtained SDF were redispersed in deionized water for further experiments.

### Synthesis of NOTA-A20

Add 400 µL of sodium carbonate/sodium bicarbonate buffer (pH = 9.2) to a 100 µL solution of NH_2_-A20 (8.2 nmol) in water and mix thoroughly. Weigh 0.23 mg of p-SCN-Bn-NOTA (M: 559.9) powder and dissolve it completely in 10 µL anhydrous DMSO, minimizing air exposure to prevent moisture absorption or direct contact with water. In the reaction system, add 10 µL of p-SCN-Bn-NOTA solution (410 nmol) to the NH_2_-A20 solution in a 50:1 molar ratio (p-SCN-Bn-NOTA: NH_2_-A20), mixing rapidly. Conduct the reaction in the dark, shaking at room temperature for 2 h. Purify the reaction product using a PD-10 column.

### Radiolabeling of DNA frameworks (ssDNA, TDF, BDF, SDF)

10 µL of NOTA-A20 (3 nmol) was added to 300 µL, 74 MBq (2 mCi) of ^68^GaCl_3_ (NaOAc buffer, 0.1 M, pH 7.5) at 37 °C and reacted for 30 min with continuous shaking. The reaction process was monitored in real time using radio thin layer chromatography (radio-TLC). The reaction mixture was purified by a PD-10 column to obtain ^68^Ga-A20, which was then mixed and hybridized with TDF (molar ratio of ^68^Ga-A20/ TDF was 4:1 in PBS buffer, pH 7.0) or BDF (molar ratio of ^68^Ga-A20/ BDF was 3:1 in PBS buffer, pH 7.0) resulting in ^68^Ga-TDF or ^68^Ga-BDF.

37 MBq (1 mCi) of ^68^GaCl_3_ or ^64^CuCl_2_ was added to 300 µL of sodium acetate buffer (0.05 M, pH = 4.0), followed with the addition of 200 µL of SDF (in deionized water). After shaking for 15 min, ^68^Ga-SDF or ^64^Cu-SDF could be obtained after PD-10 desalting with PBS as the mobile phase. Radiolabeling yields were quantified by using radio-thin layer chromatography (radio-TLC).

### Characterizations (gel electrophoresis, DLS, TEM)

Polyacrylamide gel electrophoresis (PAGE) was used to characterize the A20, TDF, BDF. DNA samples were separated on 8% PAGE gels (running buffer: 1× TAE with 12.5 mM MgAc_2_) and run for 90 min at 4 °C. The gels were then stained with GelRed and imaged under imaging system (UVP GelStudio PLUS Touch, Germany).

Inject the appropriately diluted SDF sample into the sample chamber, ensuring that the sample is uniformly suspended and free from precipitates or large particles. Avoid introducing air bubbles, maintain constant temperature and pressure conditions, initiate particle size measurement, and subsequently perform ZETA potential measurements on the same sample.

Take an appropriate volume of the SDF sample solution and place it in a centrifuge tube. Vortex the solution thoroughly. Using specialized curved-tip tweezers, extract a copper grid from the sample box and place it on filter paper. Utilize a micropipette (10 µL) to draw a small amount of the sample and carefully drop it onto a carbon support film. Prepare a uranium acetate solution with a concentration of 2.00%. After 30 min, add an appropriate amount of the uranium acetate solution to the same position on the copper grid. After 1 min, absorb excess uranium acetate solution with filter paper. Allow the sample to air-dry in a suitable environment (free from vibrations, protected from light) before transferring it to TEM for observation.

### PET imaging of DNA frameworks in healthy mice (Ga-68 and Cu-64)

Approximately 100 µL (3.7 Mbq) of ^68^Ga-A20 (^68^Ga-TDF, ^68^Ga-BDF) was administered intravenously to mice (*n* = 3) to monitor the biodistribution of A20 (TDF, BDF) in vivo. PET/CT imaging was performed at three time points of 0.5, 1 and 2 h after injection. Approximately 100 µL (3.7 Mbq) of ^68^Ga-SDF or ^64^Cu-SDF was administered intravenously to mice (*n* = 3) to monitor the biodistribution of SDF in vivo. PET/CT imaging was performed at three time points of 0.5, 1 and 2 h after injection of ^68^Ga-SDF. ^64^Cu-SDF in animals was imaged longitudinally at 15 min, 3, 12, and 24 h after injection. Region-of-interest (ROI) analysis of decay-corrected whole-body PET images was done for major organs using Inveon Research Workplace (IRW). Tracer uptake in percentage of injected dose per gram of tissue (%ID/g) and time-activity curves were charted accordingly. At the final time point after PET/CT scanning, all mice were euthanized and dissected to collect major organs/tissues for biodistribution measurement and analysis.

### Fluorescence imaging of SDF

5 µL of 100 µM Cy5-T20 was vortex-mixed with the synthesized SDF, and the reaction was centrifuged and washed three times after 10 min to obtain Cy5-SDF, which was injected into mice via tail vein and sacrificed for fluorescence imaging of organ pairs at the 0.5, 1, and 2 h time points and quantitatively analyzed.

### SDF surface protein assay analysis

SDF and serum were mixed in the ratio of 20% nano, 80% serum (100% pure mouse serum), and the reaction was shaken at 37 °C for 4 h. The supernatant was discarded and the sample was washed twice with ultrapure water and collected, and sent for mass spectrometry analysis.

### BioTEM imaging of the liver tissues

Sacrificed mice were dissected within 1–3 min, and the liver and kidneys were promptly excised. The extracted liver and kidney tissues were then rapidly processed to obtain thin, rice-grain-sized specimens, minimizing mechanical damage and bruising during tissue retrieval. Immediately after collection, the tissues were immersed in electron microscopy fixative at room temperature for a 2 h fixation period. Subsequently, the specimens were transferred and stored at 4 °C. Further processing included dehydration, embedding, curing, sectioning, staining, and final observation through TEM.

### Establishment of hepatic ischemia reperfusion injury models

All animal studies were performed under protocols approved by the Huazhong University of Science and Technology Institutional Animal Care and Use Committee. Pre-operative disinfection of the abdomen of mice was done and a midline incision was made to expose the portal triad in the liver after moving intestines towards the left side of the abdominal cavity and covered with moist drapes. Then, all structures in the portal triad (hepatic artery, bile duct, and portal vein) to the left and median liver lobes were blocked with a microvascular clamp for 60 min under anesthesia, followed with removal of the clamp to induce blood reperfusion. After verifying absence of abnormal signs, intestines were carefully replaced into the peritoneal cavity and abdomen was then sealed layer by layer with sterile medical silk sutures. In the sham group, the liver was exposed under the same surgical procedure without the ligation and reperfusion process. Blood and liver tissues were harvested for further analysis at 12 and 72 h after model establishment.

### ROS scavenging activity assay

Two main ROS, •O_2_^−^, •OH, were used to evaluate the ROS scavenging capability of M13 and SDF. All experiments were conducted according to the protocols of different assays. The superoxide anion scavenging activity was conducted with a SOD assay kit (Sigma-Aldrich, USA). Scavenging activity of hydroxyl radical was measured with a hydroxyl radical antioxidant capacity (HORAC) assay kit (Cell Biolabs, Inc., USA).

### Cytotoxicity assay of the SDF

The cytotoxicity of the SDF and M13 were detected using Cell Counting Kit-8 (CCK-8). MIHA cells were cultured in Duchenne’s Modified Eagle’s Medium (DMEM) supplemented with 10% fetal bovine serum (FBS). The cells were stored in a humidified incubator at 37 °C with 5% CO_2_. After seeding 5000 cells per well in a 96-well plate and culturing them overnight, 10 µL of a certain concentration of the SDF or M13 was added to each well and incubated for 24 h at 37 °C. Then, 10 µL of CCK-8 solution was added to each well and incubated at 37 °C for 4 h. Absorbance values were measured at 450 nm using a microplate reader (Biotek Epoch, USA).

### Pharmacokinetics study

The healthy mice were injected with 3.7 MBq of ^68^Ga-SDF via the tail vein. Blood samples were collected from the retro-orbital vein of mice with capillary tubes at multiple time points (1,3,5,10,15,20,30,45,60,90,120,160,180 min). They were weighted, and the radioactivity was measured by a gamma counter (2470, WIZARD; PerkinElmer, Waltham, MA). The plasma concentration of the drugs was presented in terms of MBq/ and analyzed by a two-compartmental model after attenuation correction. The plasma concentration versus time curves were drawn by the GraphPad Prism software2 (version 9.5, USA).

### SDF Biotoxicity Analysis

Healthy mice were injected with the drug and a group was taken whole blood and tissue organs after 12 h. Whole blood was used for analysis of complete blood cell counts, serum was used for determination of indices of liver and kidney function, and heart, liver, spleen, lung, and kidney were used for HE staining to determine organ damage by SDF. One group was continuously observed for weight changes.

### Treatment of hepatic injury

For treatment of IRI mice, SDF (1 mg/kg), M13 (1 mg/kg) or PBS (1x) were injected into the mice (*n* = 5). The mice in the sham group and healthy mice treated with the SDF (1 mg/kg), M13(1 mg/kg) or PBS (1x) were used as a control group (*n* = 5). After 12 h of the hepatic IRI model induction, their liver function was evaluated. For a long-term assessment of liver function, one group of IRI mice treated with SDF (*n* = 5) were sacrificed at 72 h after treatment.

### Liver function test and H&E staining

Liver function test and hematoxylin and eosin (H&E) staining were performed to assess the treatment of IRI. Mice in all groups were sacrificed, and blood samples were collected into lithium heparin tubes (BD Biosciences, San Jose, CA, USA) and centrifuged (2000 g at 4ºC) for 15 min to obtain the serum for analysis of alanine aminotransferase (ALT) levels and aspartate aminotransferase (AST) levels. Livers were also collected at 12 h after the model induction and fixed with paraformaldehyde (4% in PBS) for sectioning and H&E staining.

### Antioxidant indices detection after treatment

Livers in each group were frozen and stored at -80℃ until testing was performed. Liver homogenates were prepared according to different experimental schemes. SOD levels and DNA damage were measured and evaluated using the SOD assay kit and the DNA damage competitive ELISA kit (Invitrogen, USA) respectively. The degree of lipid peroxidation was evaluated with a TBARS assay kit (Cayman Chemical, USA).

### Immunofluorescence staining

Frozen slides containing liver sections were warm up at room temperature with fresh PBS, the slides were then fixed by passing through a 4% PFA solution for 10 min. By two washings with PBST (PBS with 0.2% Triton), liver sections were further permeabilized in PBS containing 2% Triton for 15 min. After PBST washing, samples were blocked using a blocking buffer (PBS including 5% horse serum and 0.3% Triton) for 1 h. Antibodies including anti-mouse F4/80, CD31, Ly6G (Biolegend), CLEC4F (R&D System) and Caspase 3 (Abcam) were diluted in blocking buffer by different combinations and incubated with liver sections overnight at 4 °C. After that, slides were washed and incubated with blocking buffer with secondary antibodies with fluorescence Alexa Fluor 488, 594 and 647 for one more hour. Finally, the liver sections were mounted using the vectashield mounting medium (Vector Laboratories, Burlingame, CA, USA). Slides were further examined and the confocal images were obtained via a confocal microscope.

### Flow cytometry analysis

Macrophage RAW264.7 cells were analyzed by flow cytometry for each cohort. The macrophage RAW264.7 was subjected to hypoxia for 12 h and reoxygenation for 6 h to create an in vitro HIRI model. After obtaining single cell suspensions, cells were incubated with fluorophore-conjugated antibodies against the following surface markers: mouse CD11c-PE/Cy7 (BD, clone: HL3), CD11b-KO525 (BD, clone: M1/70). Fixable Viability Stain510 (BD, Cat# 564,406) eliminated dead cells.

For flow cytometry of tissue infiltrating cells, the separated liver was fully cut and ground, Liver tissue was gently ground and washed with 1640 medium for further cell collection, red blood cells were lysed with red blood cell lysis buffer (Beyotime, Cat# C3702), and single cells were harvested after filter. Cell suspensions were incubated with fluorophore-conjugated antibodies against the following surface markers: mouse CD45-FITC (BD, USA, clone: 104), Ly6C-PE (BD, clone: AL-21), F4/80-PB450 (BD, clone: T45-2342), and CD11b-KO525 (BD, clone: M1/70). Dead cells were eliminated using Fixable Viability Stain510 (BD, Cat# 564,406). All surface markers were stained at 4 ℃ for 30 min in the dark. After washing, the cells were resuspended with FACS buffer and filtered through a 200-mesh cell strainer, followed by flow cytometry using a CytoFLEX machine. CytExpert software (Version 2.4) was used for Flow cytometry analysis; the gating strategy for each cell population is shown in Fig. S2a (online).

### Cytokines measurements by enzyme-linked immunosorbent assay (ELISA)

Obtained livers were cut into small pieces, and homogenized in PIPA buffer (Boston Bio Products) containing 1 x protein inhibitor (Pierce Protease inhibitor tablets, Thermofisher) at a final concentration of 200 mg/mL. All processes were conducted on ice. Then, lysates were obtained after 20,000 g centrifugation for 20 min at 4 °C and stored in -80℃ until use. Before tests, samples were thawed on ice and diluted in a serial of dilutions (1:10 to 1:640). The following measurements of secreted cytokines by ELISA were conducted according to the manufacturer’s instructions (Mouse IL-1 ELISA development Kit, PromoKine; Mouse TNF-α ELISA Kit, Cayman; Mouse IL-12, IFN-γ and IL-6 ELISA Kit, Bioleagend; Mouse Myeloperoxidase ELISA Kit, R&D system; Mouse NOS2 ELISA Kit, G-Bioscience).

### In vivo toxicity assessment

150 µL (1 mg/kg) of SDF was intravenously injected into C57BL/6 mice (*n* = 4) and the same dose of normal saline was injected into another group of mice as control group. Blood samples and organs were collected from both groups 72 h after injection for H&E staining to monitor the histological changes of the heart, liver, spleen, lungs, and kidneys. After measured complete blood panel data from the experimental and control groups. The blood samples were then centrifuged at 2,000 g for 15 min at 4ºC to obtain the plasma for analysis of liver and kidney functional profiles.

### Electronic supplementary material

Below is the link to the electronic supplementary material.

Supplementary Material 1

## Data Availability

All data is available in the main text or the supplementary materials.
