# Supplementary material for: Liver-specific delivery of spherical DNA frameworks for alleviation of hepatic ischemic reperfusion injury
Source: J Nanobiotechnology. 2024 Jul 4;22:394. doi: 10.1186/s12951-024-02661-8 (PMC11223400; doi:10.1186/s12951-024-02661-8)
Supplement: Supplementary file 1 — Supplementary Material 1 [file 12951_2024_2661_MOESM1_ESM.docx]

**Liver-specific delivery of spherical DNA frameworks for alleviation of hepatic ischemic reperfusion injury**

Hao Wang^1,3,4#^, Li Wen^1,3,4#^, Hao Wei^1,3,4^, Yangmeihui Song^1,3,4^, Wenyu Song^1,2,3,4^, Mengting Li^1,3,4^, Xiaoli Lan^1,3,4*^, Weibo Cai^2*^, Dawei Jiang^1,3,4*^

1. Department of Nuclear Medicine, Tongji Medical College, Huazhong University of Science and Technology, Wuhan, 430022, China

2. Departments of Radiology and Medical Physics, Madison, WI, 53705, USA

3. Hubei Key Laboratory of Molecular Imaging, Wuhan, 430022, China

4. Key Laboratory of Biological Targeted Therapy, Ministry of Education, Wuhan, 430022, China

# These authors contributed equally to this work

**Corresponding authors:**

Dawei Jiang, PhD, 1277 Jiefang Ave., Wuhan, 430022, China.

Email: daweijiang@hust.edu.cn

Weibo Cai, PhD, 600 Highland Ave., K6/562, Madison, WI 53792, USA

Email: wcai@uwhealth.org

Xiaoli Lan, MD PhD, 1277 Jiefang Ave., Wuhan, 430022, China

Email: xiaoli_lan@hust.edu.cn

**Fig. S1** Step-wise assembly of TDF and BDF.

**Fig. S2** Longitudinal PET imaging of ^68^Ga-TDF(A), ^68^Ga-BDF(B)in healthy mice.

**Fig. S3** Organ ROI analysis of ^68^Ga-SDF PET images in healthy mice.

**Fig. S4** Mice organs were fluorescently imaged (A) and quantified (B) by tail vein injection of SDF and sacrificing mice at 0.5, 1, and 2 h time points.

**Fig. S5** PET images of mice at various time points after i.v. injection of ^64^Cu-SDF.

**Fig. S6** Cytotoxicity assay of SDF (A) and M13 (B).

**Fig. S7** Biotoxicity assessment of SDF.

**Fig. S8** (A) Gene ontology (GO) enrichment analysis, (B) KEGG pathway enrichment analysis, (C) Surface protein analysis of SDF.

**Fig. S9** Pharmacokinetic analysis of SDF.

**Fig. S10** Analysis of the scavenging efficiencies of hydroxyl radicals (•OH; A) and superoxide radicals (•O_2_^-^; B) for M13, SDF.

**Fig. S11** Immunofluorescence staining semi quantitative data of 12 h.

**Fig. S12** Immunofluorescence staining on liver samples of 12 h. Immunofluorescence staining was performed using DAPI, anti-caspase-3 antibody, anti-CD31 antibody, anti-ICAM-1 antibody as markers of liver tissues.

**Fig. S13** Immunofluorescence staining on liver samples of 12 h. Immunofluorescence staining was performed using DAPI, anti-caspase-3 antibody, anti-CD31 antibody, anti-Ly6G antibody as markers of liver tissues from each group.

**Fig. S14** Immunofluorescence staining on liver samples of 72 h. Immunofluorescence staining was performed using DAPI, anti-CLE4F antibody, anti-F4/80 antibody, anti-CD31 antibody as markers of liver tissues from each group.

**Fig. S15** Immunofluorescence staining on liver samples of 72 h. Immunofluorescence staining was performed using DAPI, anti-caspase-3 antibody, anti-CD31 antibody, anti-ICAM-1 antibody as markers of liver tissues.

**Fig. S16** Immunofluorescence staining on liver samples of 72 h. Immunofluorescence staining was performed using DAPI, anti-caspase-3 antibody, anti-CD31 antibody, anti-Ly6G antibody as markers of liver tissues from each group.

**Fig. S17** Immunofluorescence staining semi quantitative data of 72 h.

**Fig. S18** Detection of cytokines in liver tissues.

**Fig. S19** Macrophage RAW264.7 was treated with LPS and SDF and analyzed for M1 percentage by flow cytometry.

**Fig. S20** A: Flow cytometry statistical analysis of macrophage RAW264.7 B: Flow cytometry statistical analysis of liver tissue.

**Fig. S21** Hypoxia-reoxygenation creates an *in vitro* HIRI model and the effect of SDF on M1 polarization is analyzed by flow cytometry.

**Table S1.** The sequence information of each strand of DNA that makes up the DNA frameworks.


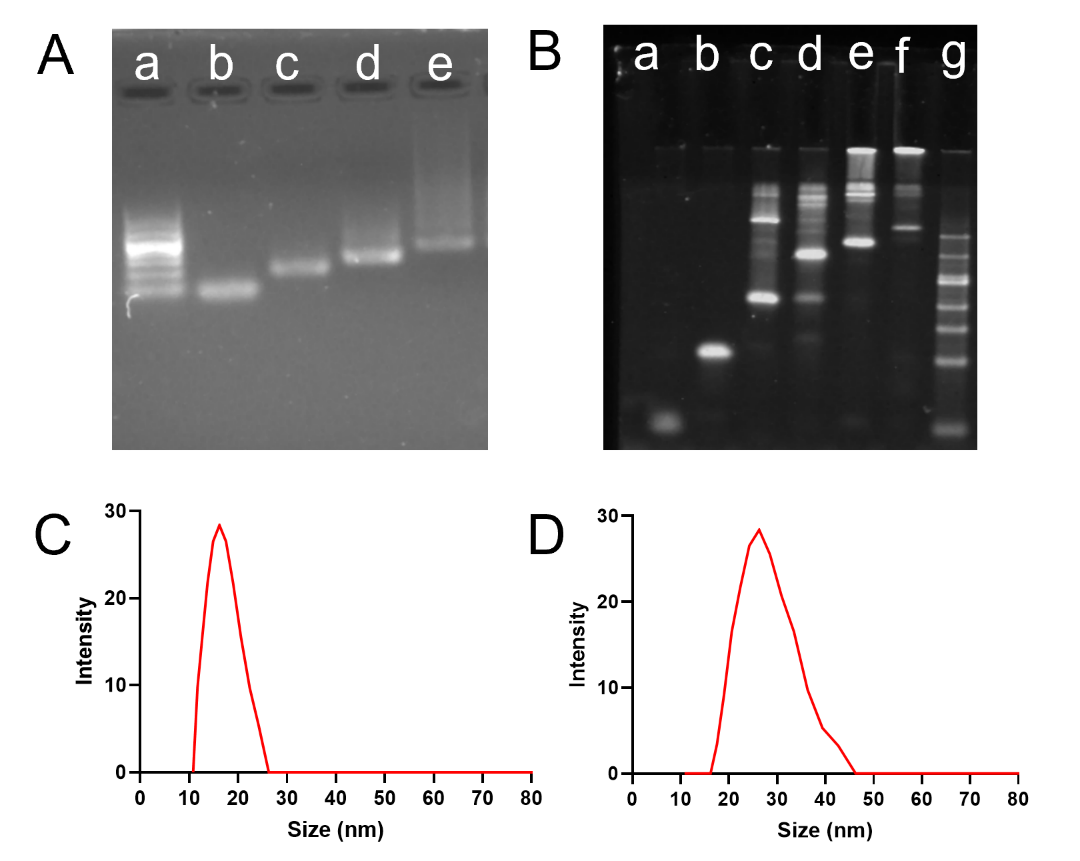


**Fig. S1** **A** Step-wise assembly of TDF (a is marker,1, 2, 3, and 4 DNA strands, respectively, corresponding to b, c, d, e lane) and characterization using polyacrylamide gel electrophoresis (PAGE). **B** Step-wise assembly of BDF (1, 2, 3, 4, 5, and 6 DNA strands, respectively, corresponding to a, b, c, d, e, f lane; g lane is marker) and characterization using PAGE. Particle size analysis of TDF(**C**), BDF(**D**).


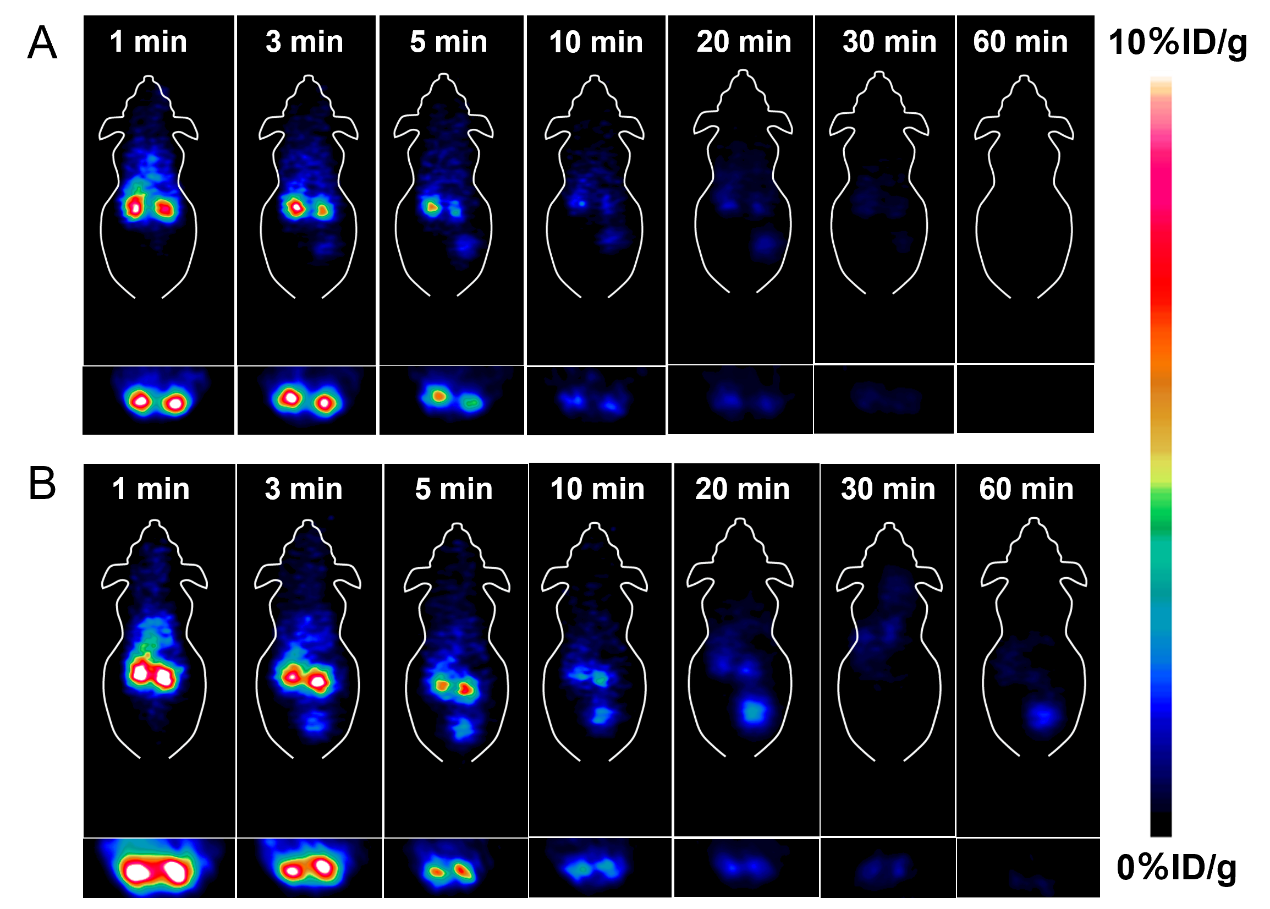


**Fig. S2** Longitudinal PET imaging (1 min, 3 min, 5 min, 10 min, 20 min, 30 min, 60 min) of ^68^Ga-TDF(**A**), ^68^Ga-BDF(**B**)in healthy mice (top, coronal slices; bottom, axial slices)


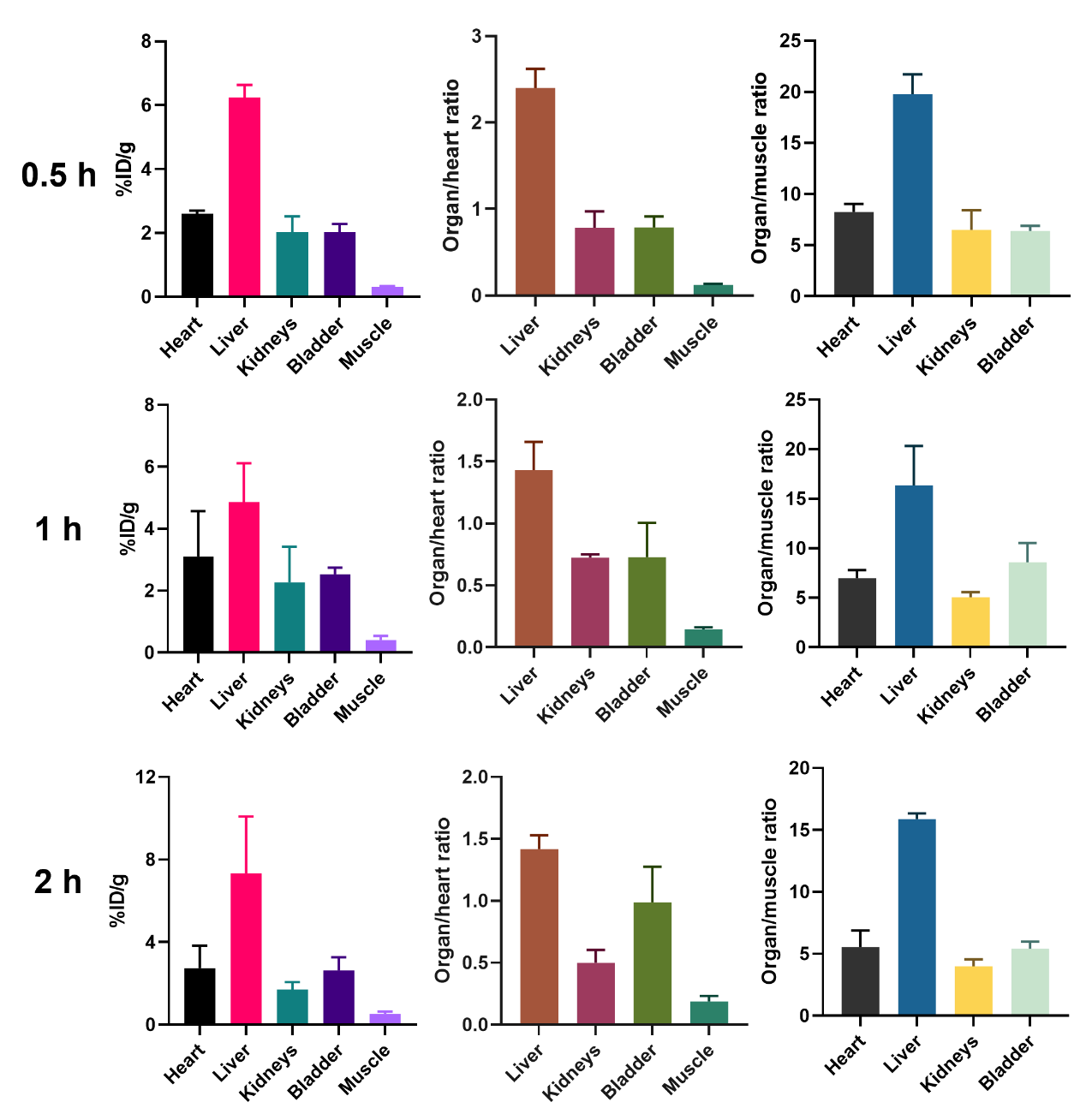


**Fig. S3** Organ ROI analysis of ^68^Ga-SDF PET images in healthy mice. Data represent means ± s.d. from three independent replicates.


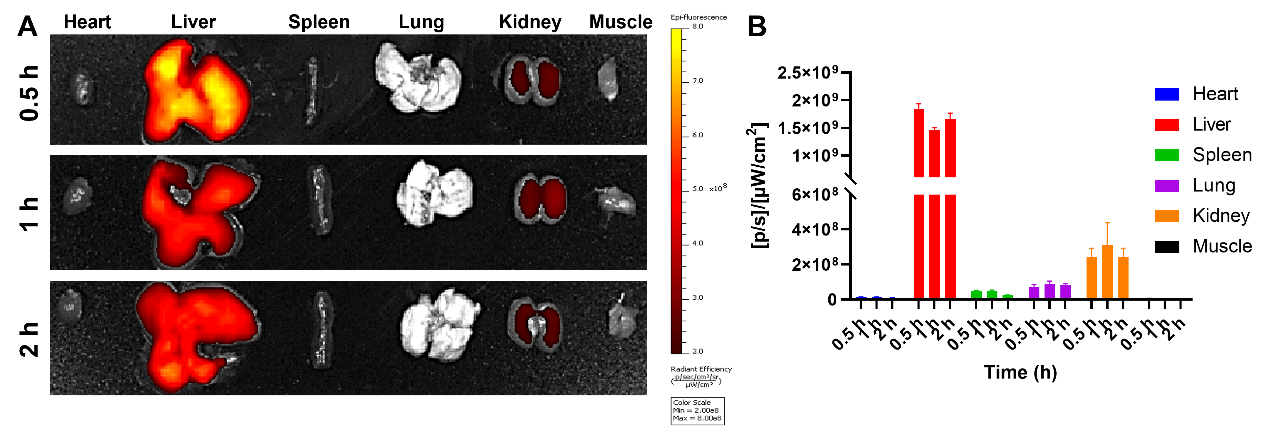


**Fig. S4** Fluorescent imaging of mice (**A**) was performed at 0.5, 1, and 2 h after tail vein injection to quantify biodistribution (**B**) of SDF *in vivo*. Data represent means ± s.d. from three independent replicates.


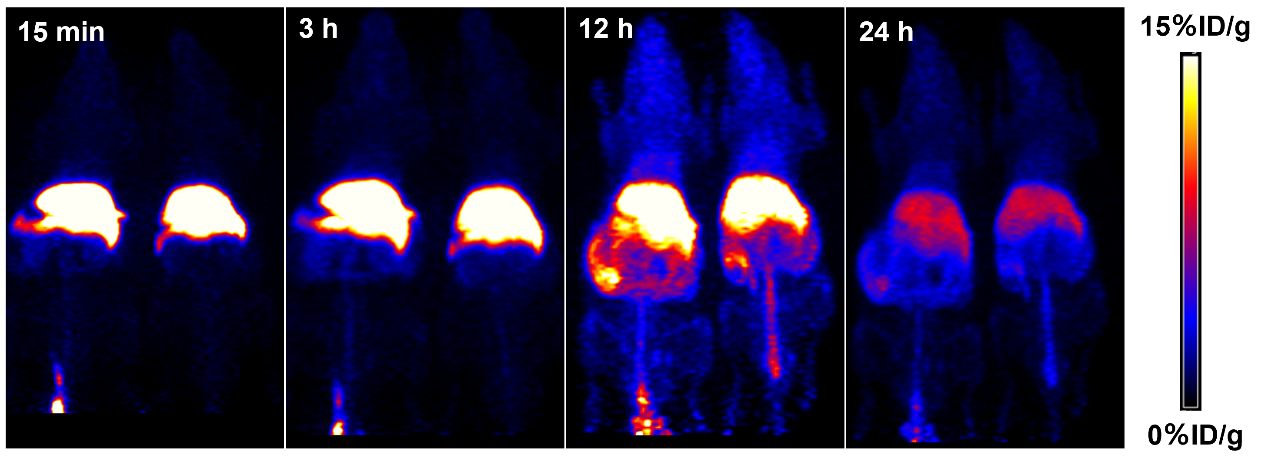


**Fig. S5** PET images (15 min, 3 h, 12 h, 24 h) of mice at various time points after i.v. injection of ^64^Cu-SDF.


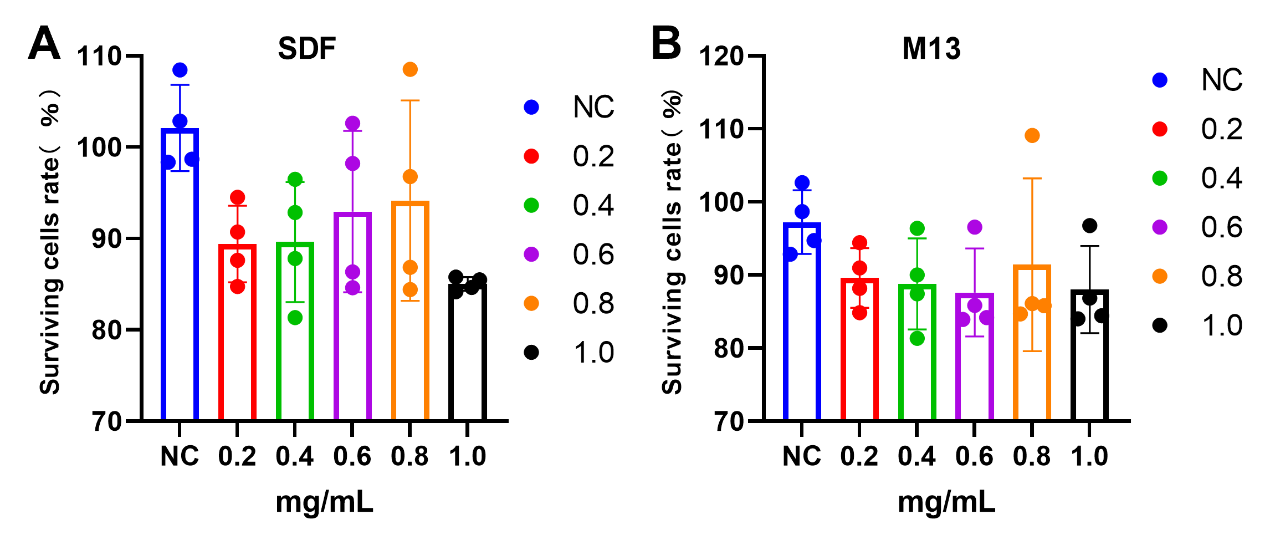


**Fig. S6** Cytotoxicity assay of SDF (**A**) and M13 (**B**).


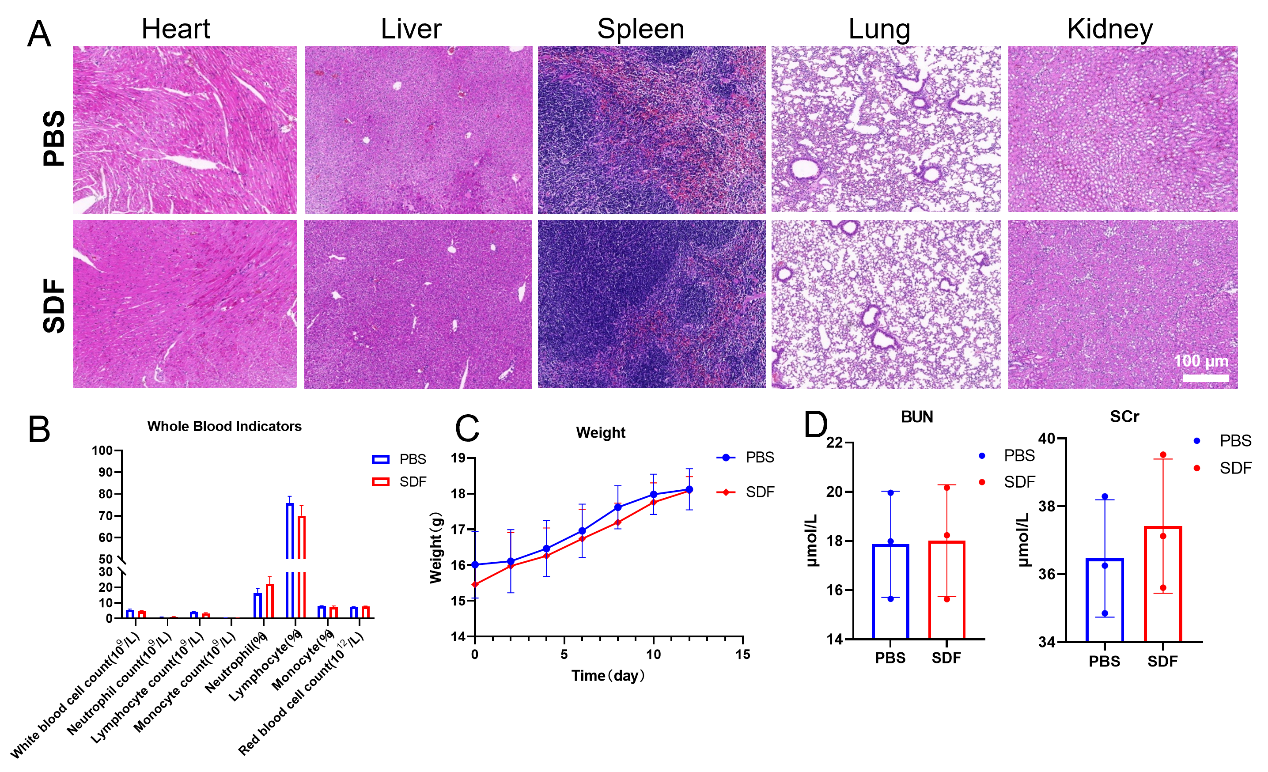


**Fig. S7** Biotoxicity assessment of SDF. **A**: H&E staining of heart, liver, spleen, lung and kidney **B**: complete blood count **C**: change in body weight of mice **D**: serum creatinine (SCr), blood urea nitrogen levels (BUN).


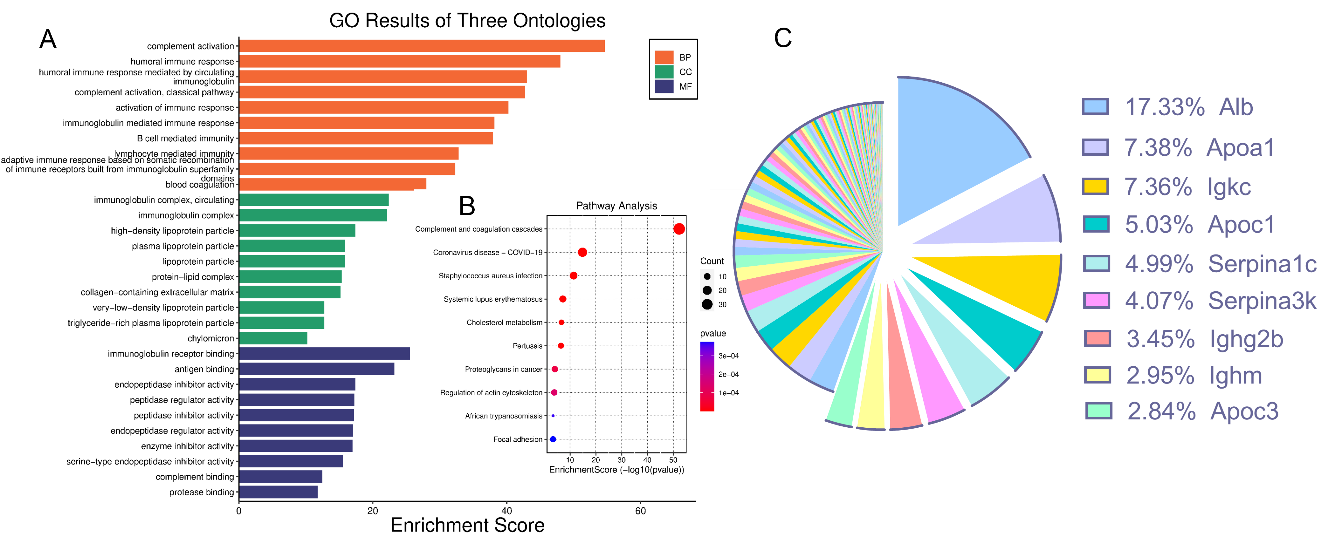


**Fig. S8** (**A**) Gene ontology (GO) enrichment analysis for key targets (top 10 were listed). (**B**) KEGG pathway enrichment analysis of key targets (top 10 were listed); the abscissa label represents Fold Enrichment of pathways. (**C**) Surface protein analysis of SDF.


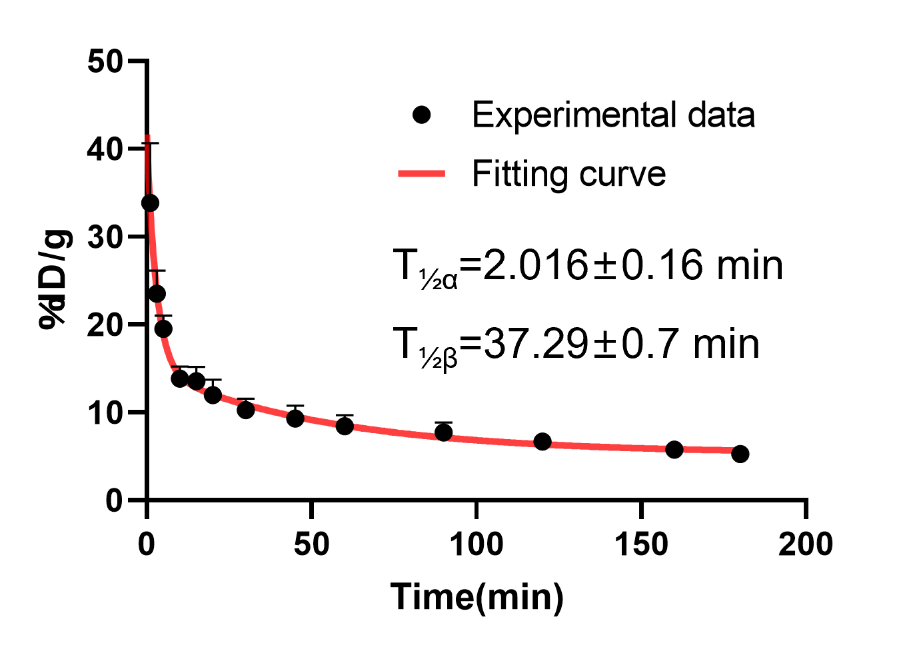


**Fig. S9** Pharmacokinetic analysis of SDF.


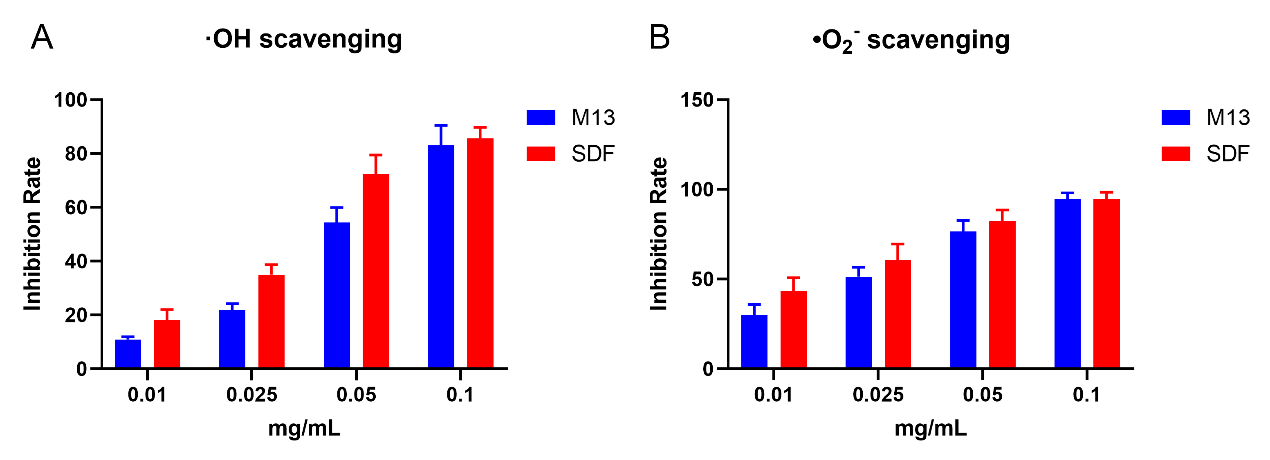


**Fig. S10** Analysis of the scavenging efficiencies of hydroxyl radicals (•OH; **A**) and superoxide radicals (•O_2_^-^; **B**) for M13, SDF. Data represent means ± s.d. from three independent replicates.


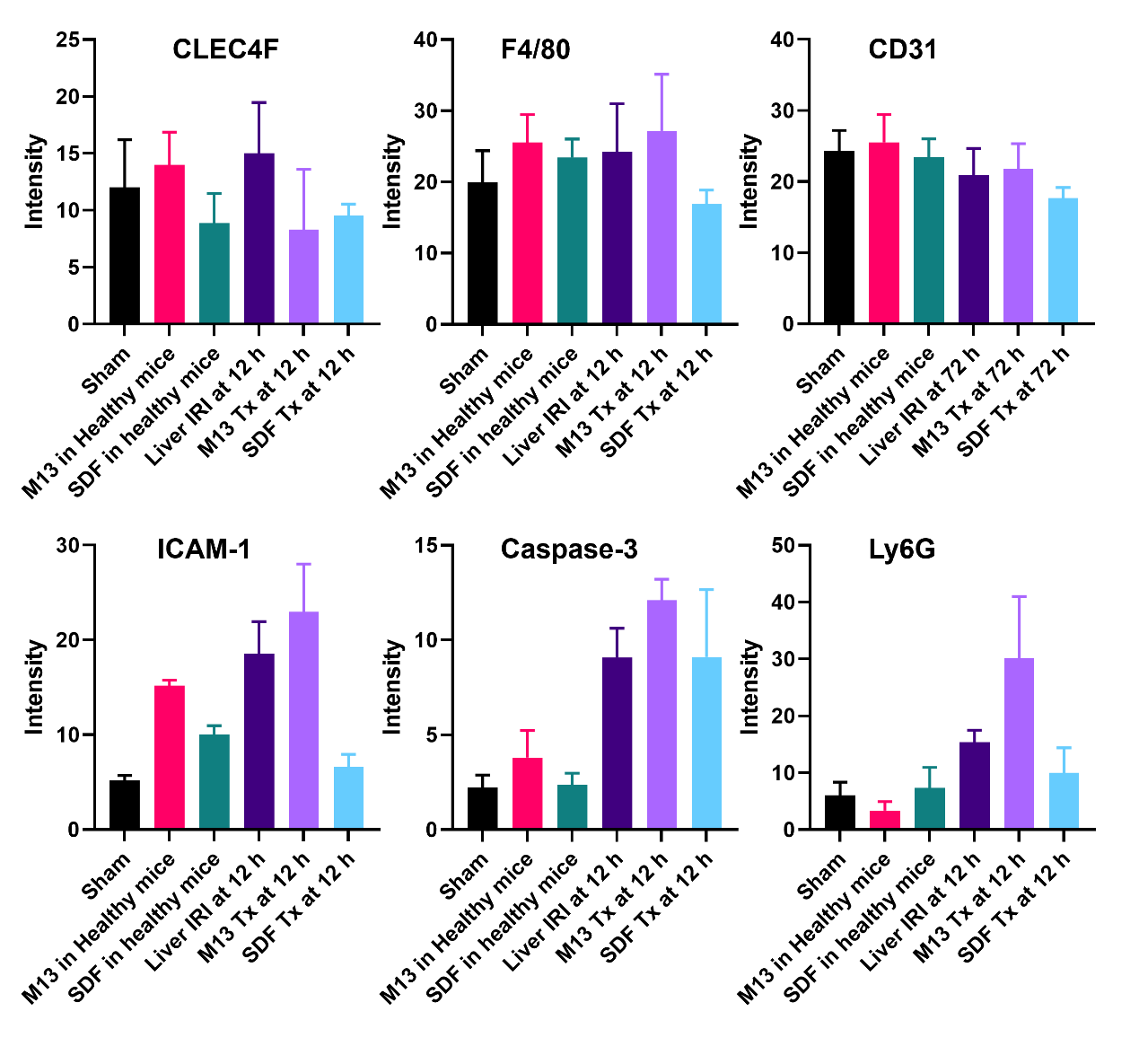


**Fig. S11** Immunofluorescence staining (CLEC4F, F4/80, CD31, ICAM-1, Caspase-3, Ly6G) semi quantitative data of 12 h. Data represent means ± s.d. from three independent replicates.


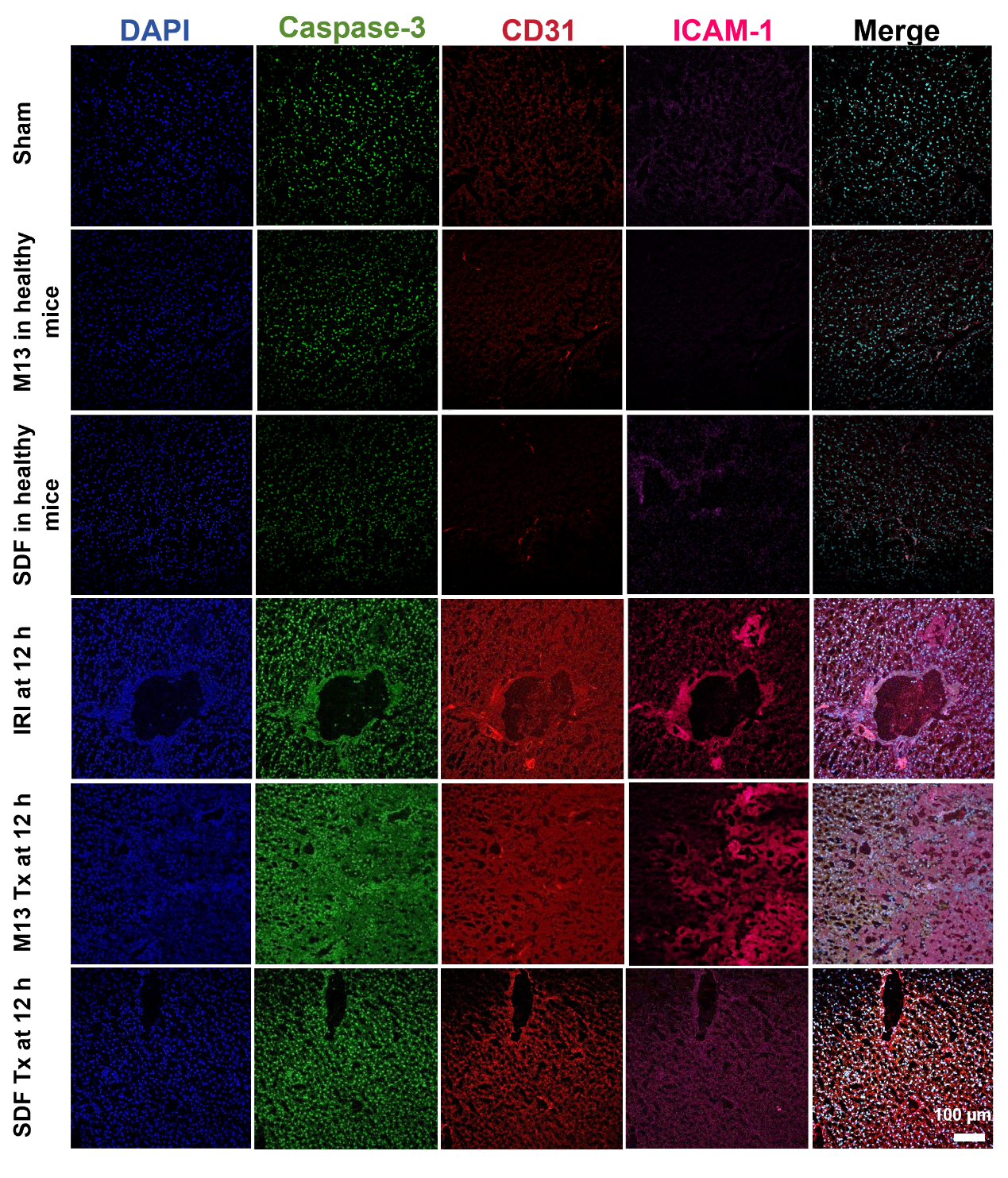


**Fig. S12** Immunofluorescence staining on liver samples. Immunofluorescence staining was performed using DAPI (blue) for nuclear staining, anti-caspase-3 antibody (green) as a cell apoptosis marker, anti-CD31 antibody (red) as an endothelial marker, anti-ICAM-1 antibody (pink) as marker of intracellular adhesion of liver tissues from each group. Scale bar: 100 µm.


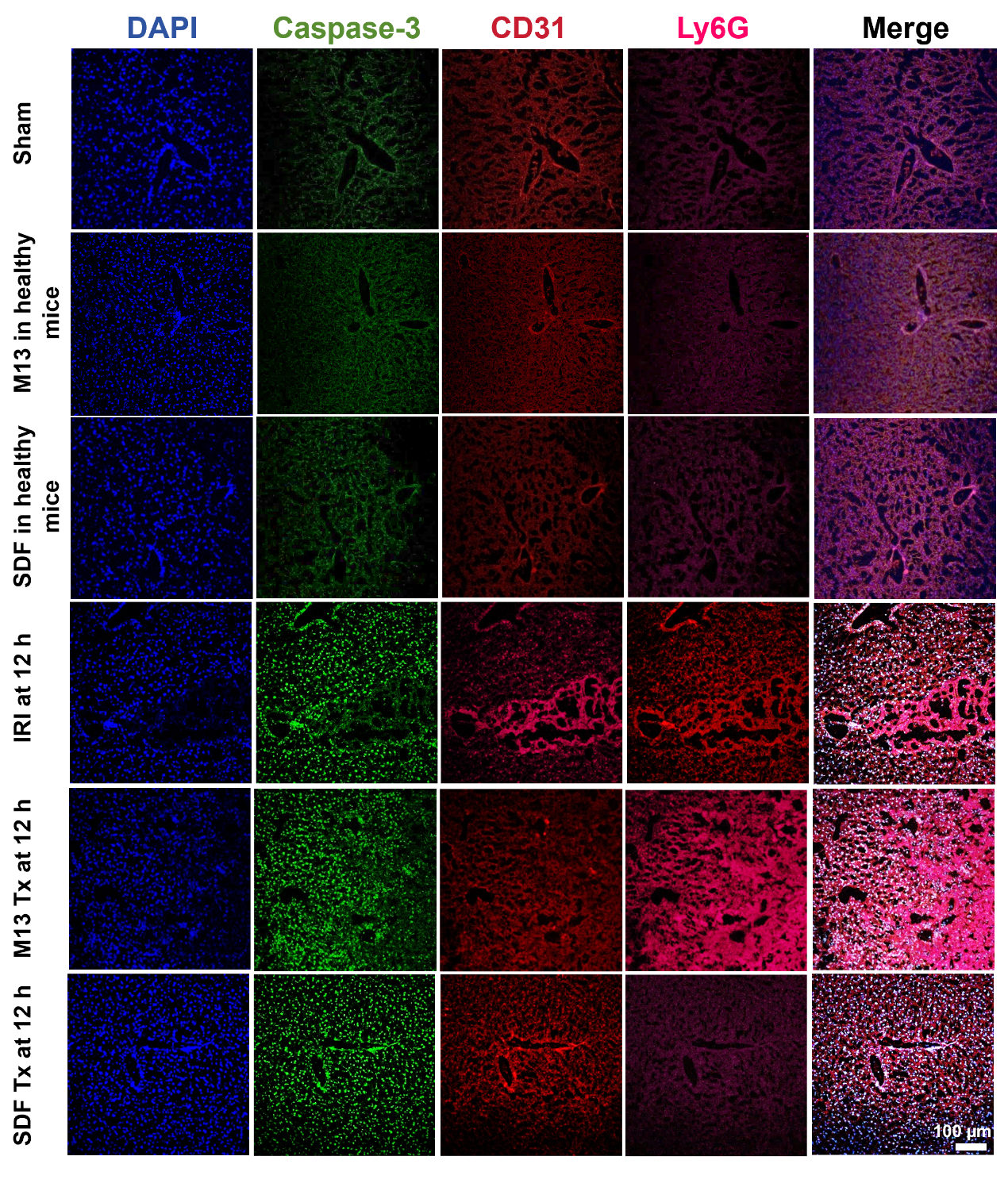


**Fig. S13** Immunofluorescence staining on liver samples. Immunofluorescence staining was performed using DAPI (blue) for nuclear staining, anti-caspase-3 antibody (green) as a cell apoptosis marker, anti-CD31 antibody (red) as an endothelial marker, anti-Ly6G antibody (pink) as marker of neutrophil of liver tissues from each group. Scale bar: 100 µm.


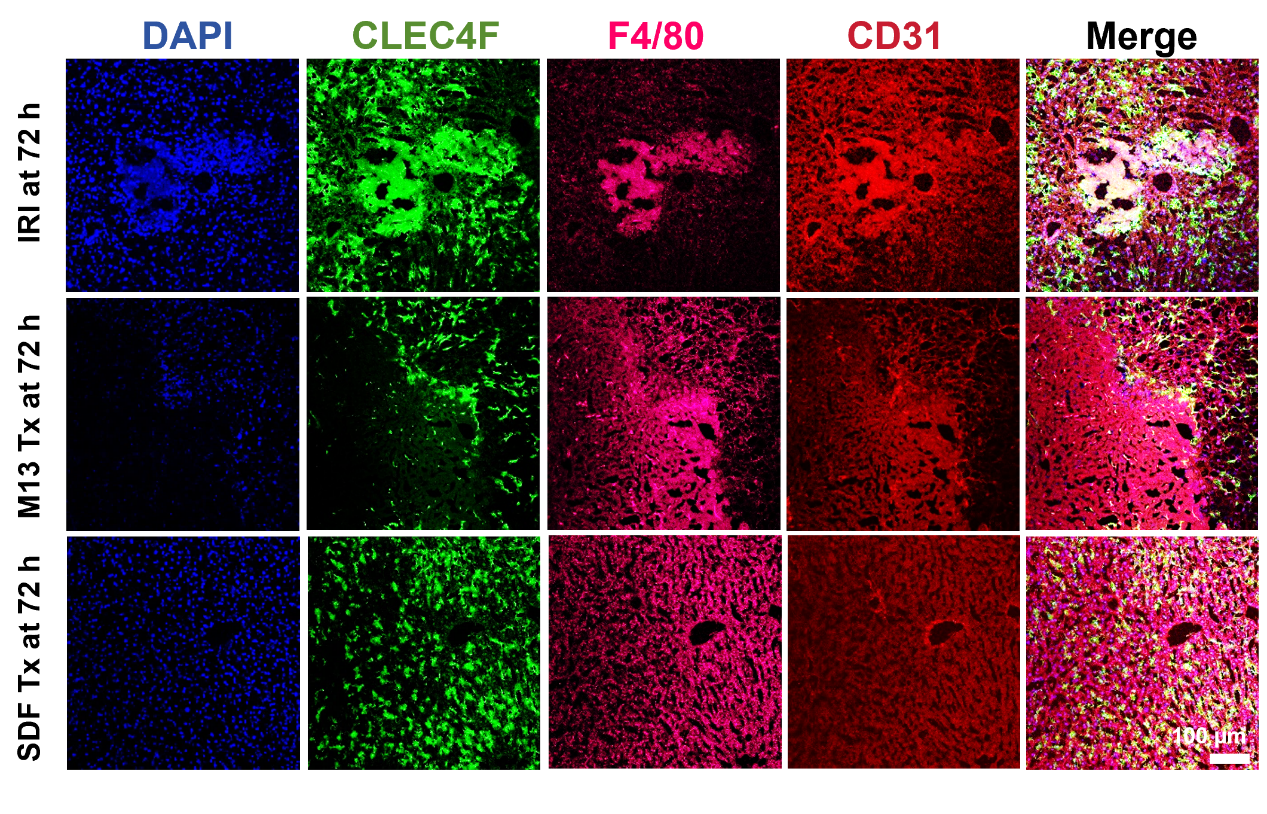


**Fig. S14** Immunofluorescence staining on liver samples of 72 h. Immunofluorescence staining was performed using DAPI (blue) for nuclear staining, anti-CLE4F antibody (green) as Kupffer cell marker, anti-F4/80 antibody (pink) as monocyte/macrophage marker, anti-CD31 antibody (red) as an endothelial marker of liver tissues from each group. Scale bar: 100 µm.


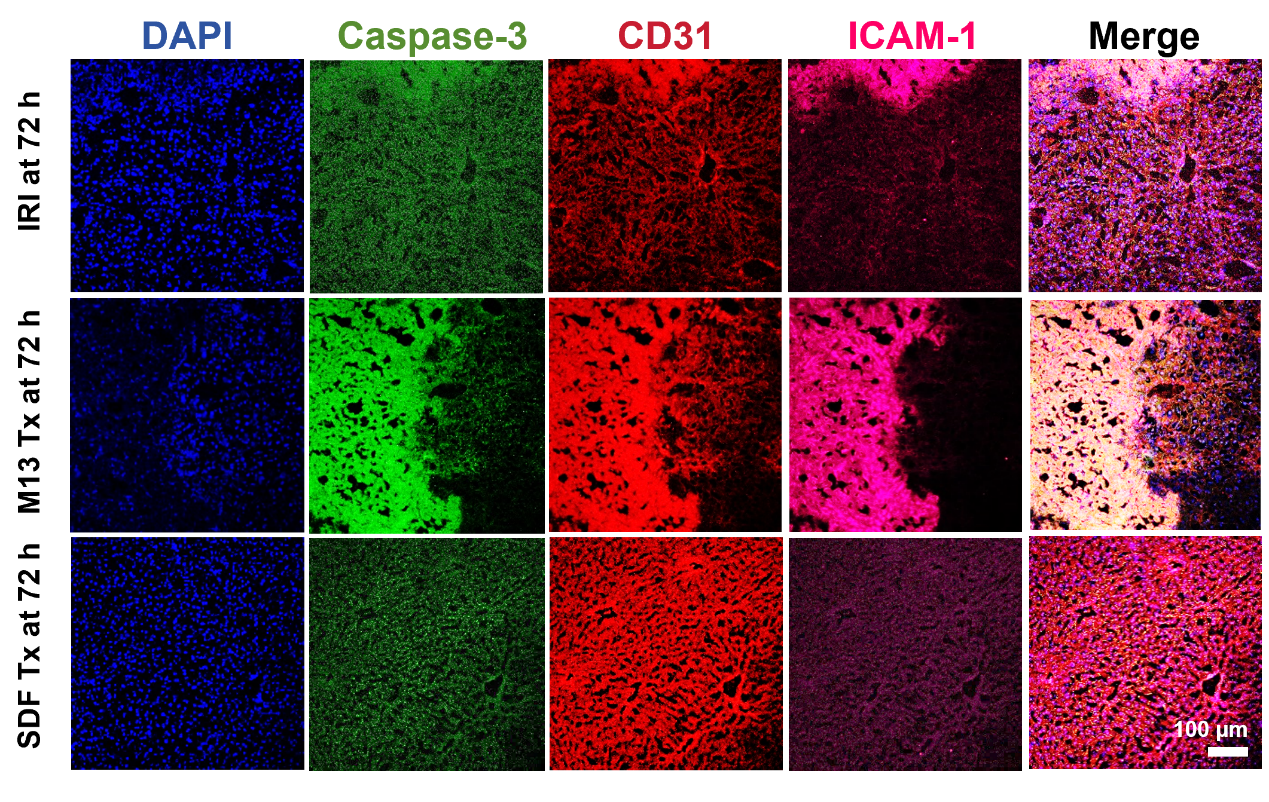


**Fig. S15** Immunofluorescence staining on liver samples of 72 h. Immunofluorescence staining was performed using DAPI (blue) for nuclear staining, anti-caspase-3 antibody (green) as a cell apoptosis marker, anti-CD31 antibody (red) as an endothelial marker, anti-ICAM-1 antibody (pink) as marker of intracellular adhesion of liver tissues from each group. Scale bar: 100 µm.


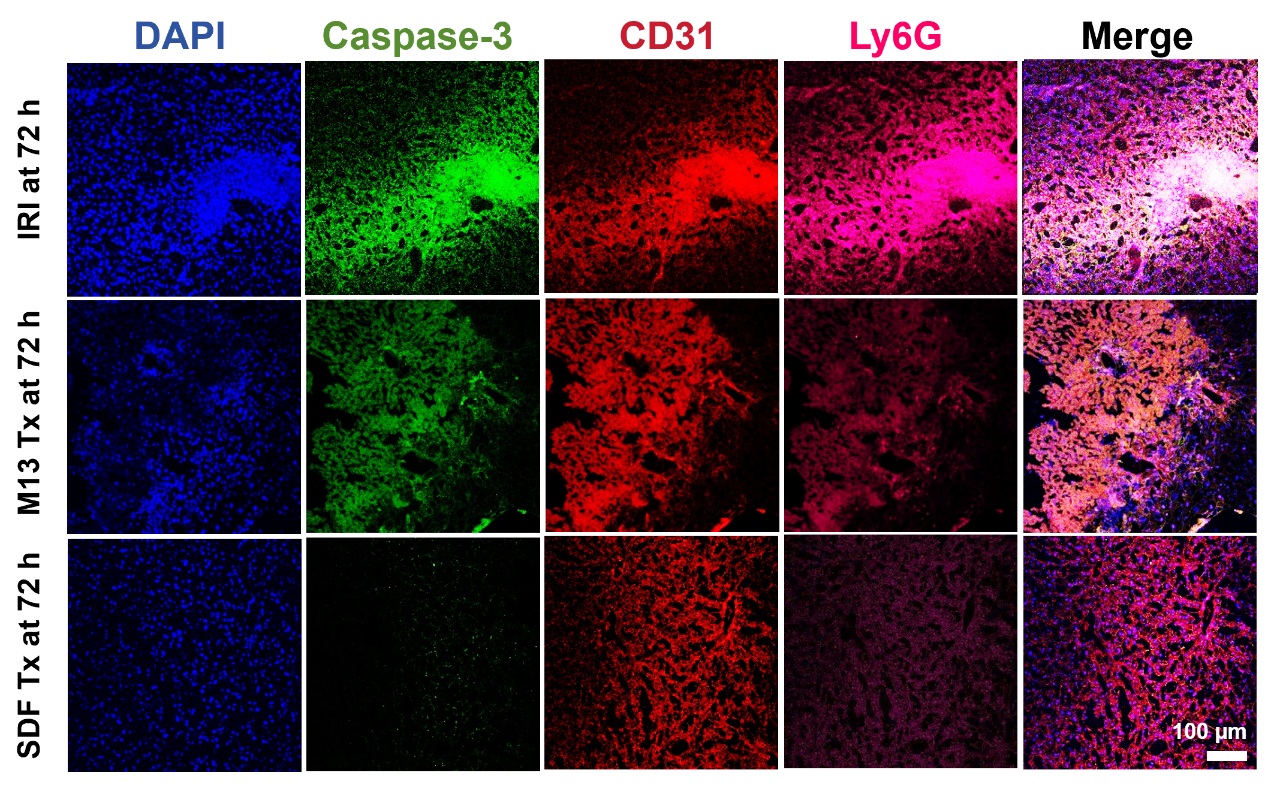


**Fig. S16** Immunofluorescence staining on liver samples of 72 h. Immunofluorescence staining was performed using DAPI (blue) for nuclear staining, anti-caspase-3 antibody (green) as a cell apoptosis marker, anti-CD31 antibody (red) as an endothelial marker, anti-Ly6G antibody (pink) as marker of neutrophil of liver tissues from each group. Scale bar: 100 µm.


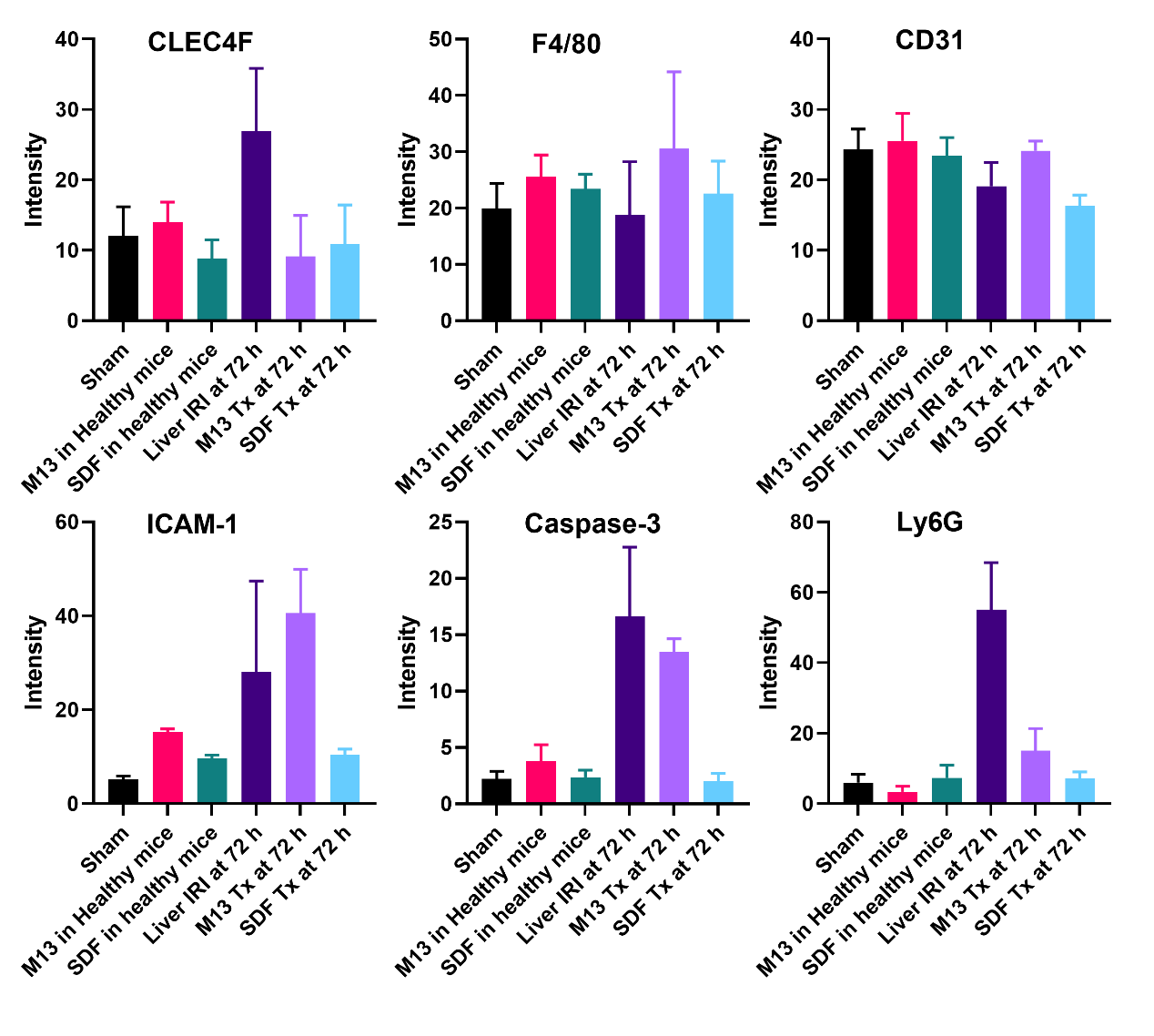


**Fig. S17** Immunofluorescence staining (CLEC4F, F4/80, CD31, ICAM-1, Caspase-3, Ly6G) semi quantitative data of 72 h. Data represent means ± s.d. from three independent replicates.


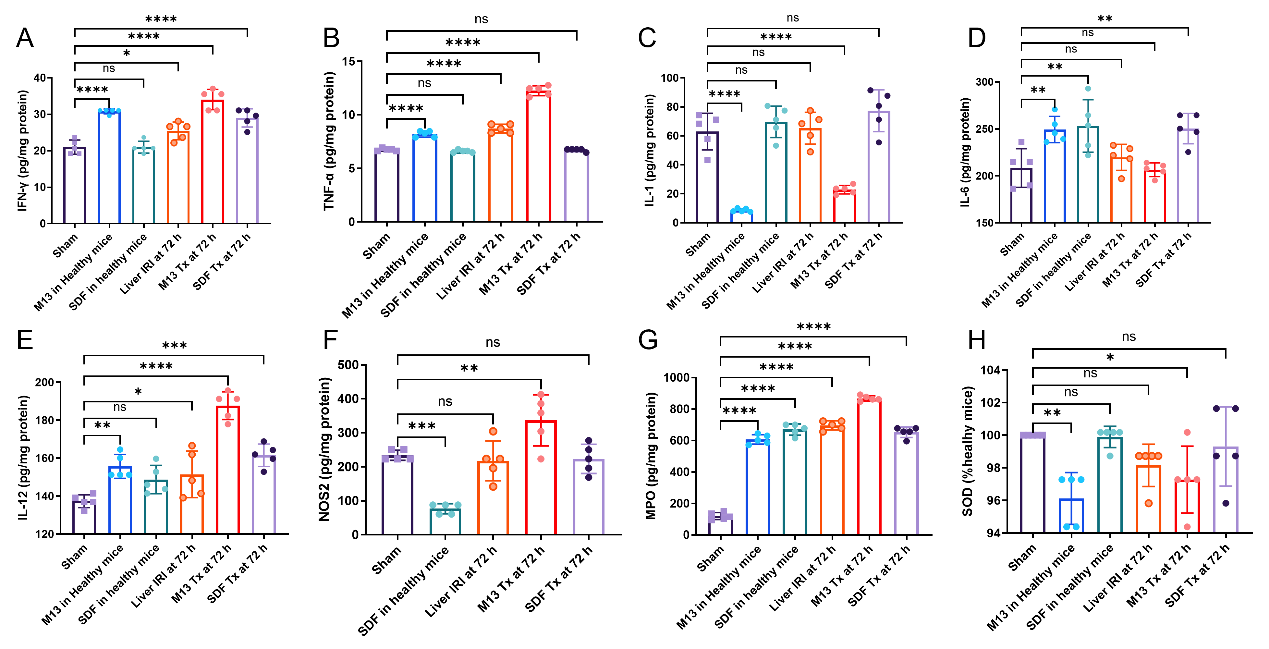


**Fig. S18** Detection of cytokines in liver tissues of 72 h. **A-H**) Cytokines of IFN-γ (**A**), TNF-α (**B**), IL-1 (**C**), IL-6 (**D**), IL-12 (**E**), and NOS2 (**F**) from activated monocyte/macrophages and Kupffer cells and MPO (**G**) from activated neutrophil were measured in liver homogenates and SOD (**H**) form each group. Data represent means ± s.d. from five independent replicates. (*P < 0.05; **P < 0.01; *** P < 0.001; **** P < 0.0001; ns, non-significant). The number of mice in each group was 5.


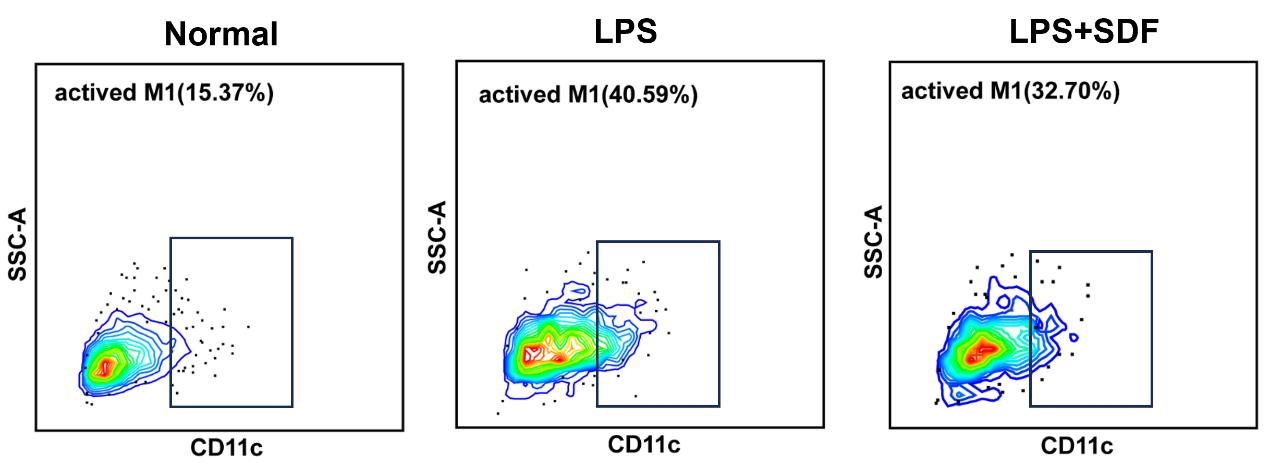


**Fig. S19** Macrophage RAW264.7 was treated with LPS and SDF and analyzed for M1 percentage by flow cytometry.


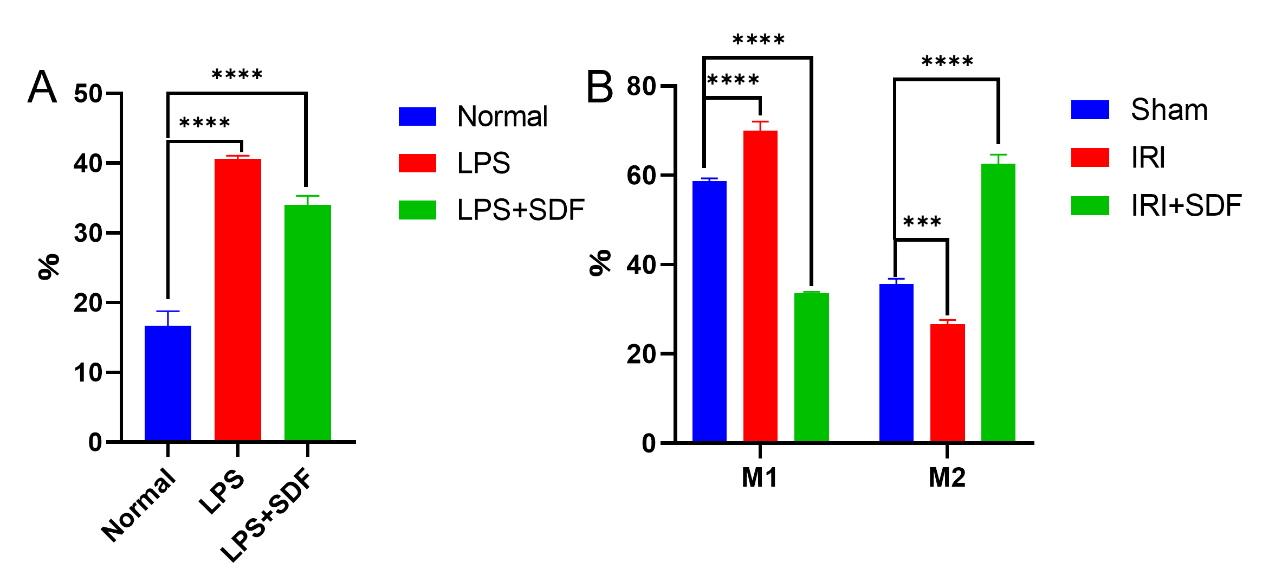


**Fig. S20 A**: Flow cytometry statistical analysis of macrophage RAW264.7 **B**: Flow cytometry statistical analysis of liver tissue. Data represent means ± s.d. from five independent replicates, and P values were calculated by one-way ANOVA with Tukey's honest significant difference post-hoc test (*P < 0.05; **P < 0.01; *** P < 0.001; **** P < 0.0001).


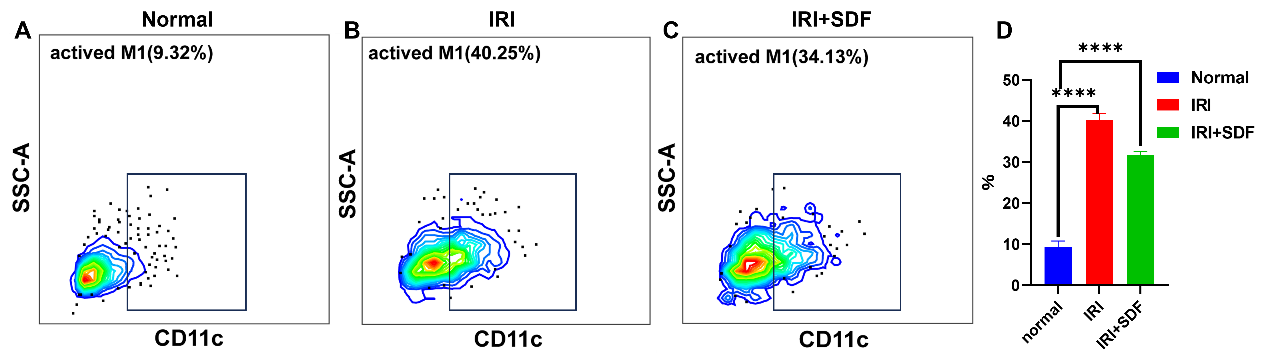


**Fig.S21** Macrophage cells RAW264.7 were treated with hypoxia-reoxygenation process to establish an *in vitro* HIRI model, and the effects of SDF on M1 were analyzed (**A**, **B**, **C**), and (**D**) shows the flow cytometry statistical analysis of macrophage RAW264.7. Data represent means ± s.d. from five independent replicates, and P values were calculated by one-way ANOVA with Tukey's honest significant difference post-hoc test (*P < 0.05; **P < 0.01; *** P < 0.001; **** P < 0.0001).

**Table S1. DNA sequence information**

| DNA frameworks | Sequence abbreviation | Sequence (from 5’ to 3’) |
| --- | --- | --- |
| A20 | A20 | AAAAAAAAAAAAAAAAAAAA |
| TDF | A | TTTTTTTTTTTTTTTTTTTTATTGCTGTATTGGCTCTGGTGATGCGTTAAAGGATCTCGTATAGCAGCTCAGTCCACTCGAAC |
|  | B | TTTTTTTTTTTTTTTTTTTTCATAGTCAATAACGCATCACCAGAGCCAAATGACGACATCTGTGCGATGAAACCTAGCAGACC |
|  | C | TTTTTTTTTTTTTTTTTTTTGAGATCCTATGACTATGGGTCTGCTAGGTACAGTCTGTCGCTTATGCACTAGAGCTGCTATAC |
|  | D | TTTTTTTTTTTTTTTTTTTTTGTCGTCAAACAGCAATGTTCGAGTGGACAAGTGCATAAGCGACAGACTGATCATCGCACAGA |
| BDF | a | TTTTTTTTTTTTTTTTTTTTCTAAGTCTGAAATTTATCACCCGCCATAGTAGACGTATCACCAGGCAGTTGAGACGAACATTC |
|  | b | TTTTTTTTTTTTTTTTTTTTCTTGCTACACGATTCAGACTTAGGAATGTTCGACATGCGAGGGTCCAATACCGACGATTACAG |
|  | c | GGTGATAAAACGTGTAGCAAGCTGTAATCGACGGGAAGAGCATGCCCATCCACTACTATGGCG |
|  | A | TTTTTTTTTTTTTTTTTTTTCTGCGCGGATGACTCAACTGCCTGGTGATACGATCTAGTCTCTACGTCAAGTAAGAACCTTAG |
|  | B | CCTCGCATGACATCCGCGCAGCTAAGGTTCAAAGTTCCTGCCGCTTCACGGACGGTATTGGAC |
|  | C | CTCTTCCCGACCGTGAAGCGGCAGGAACTTATACTTGACGTAGAGACTAGAAGGATGGGCATG |
